# DORIS: A diffusion MRI-based 10 tissue class deep learning segmentation algorithm tailored to improve anatomically-constrained tractography

**DOI:** 10.3389/fnimg.2022.917806

**Published:** 2022-09-22

**Authors:** Guillaume Theaud, Manon Edde, Matthieu Dumont, Clément Zotti, Mauro Zucchelli, Samuel Deslauriers-Gauthier, Rachid Deriche, Pierre-Marc Jodoin, Maxime Descoteaux

**Affiliations:** ^1^Sherbrooke Connectivity Imaging Laboratory (SCIL), Université de Sherbrooke, Sherbrooke, QC, Canada; ^2^Imeka Solutions Inc., Sherbrooke, QC, Canada; ^3^Athena Project-Team, Inria Sophia Antipolis-Méditerranée, Université Côte D'Azur, Nice, France; ^4^Videos & Images Theory and Analytics Laboratory (VITAL), Université de Sherbrooke, Sherbrooke, QC, Canada

**Keywords:** diffusion magnetic resonance imaging, tractography, anatomical constraints, image segmentation, machine learning

## Abstract

Modern tractography algorithms such as anatomically-constrained tractography (ACT) are based on segmentation maps of white matter (WM), gray matter (GM), and cerebrospinal fluid (CSF). These maps are generally estimated from a T1-weighted (T1w) image and then registered in diffusion weighted images (DWI) space. Registration of T1w to diffusion space and partial volume estimation are challenging and rarely voxel-perfect. Diffusion-based segmentation would, thus, potentially allow not to have higher quality anatomical priors injected in the tractography process. On the other hand, even if FA-based tractography is possible without T1 registration, the literature shows that this technique suffers from multiple issues such as holes in the tracking mask and a high proportion of generated broken and anatomically implausible streamlines. Therefore, there is an important need for a tissue segmentation algorithm that works directly in the native diffusion space. We propose **DORIS**, a DWI-based deep learning segmentation algorithm. DORIS outputs 10 different tissue classes including WM, GM, CSF, ventricles, and 6 other subcortical structures (putamen, pallidum, hippocampus, caudate, amygdala, and thalamus). DORIS was trained and validated on a wide range of subjects, including 1,000 individuals from 22 to 90 years old from clinical and research DWI acquisitions, from 5 public databases. In the absence of a “true” ground truth in diffusion space, DORIS used a silver standard strategy from Freesurfer output registered onto the DWI. This strategy is extensively evaluated and discussed in the current study. Segmentation maps provided by DORIS are quantitatively compared to Freesurfer and FSL-fast and the impacts on tractography are evaluated. Overall, we show that DORIS is fast, accurate, and reproducible and that DORIS-based tractograms produce bundles with a longer mean length and fewer anatomically implausible streamlines.

## 1. Introduction

Diffusion MRI is often used to explore structural brain connectivity using tractography. Tractography algorithms use orientation fields (Pierpaoli and Basser, 1996; Kreher et al., 2005; Peled et al., 2006; Tournier et al., 2007; Descoteaux, 2008; Jeurissen et al., 2014) to reconstruct the main white matter pathways of the brain. Moreover, tractography algorithms are mostly based on tracking masks to guide the algorithm where it is allowed to go and where it should stop. The easiest way to obtain a tracking mask is by thresholding the DTI fractional anisotropy (FA) map (Côté et al., 2013; Farquharson et al., 2013; Chamberland et al., 2014; Jeurissen et al., 2019; Vanderweyen et al., 2020), and this is usually recommended for pathological brains (Theaud et al., 2019; Vanderweyen et al., 2020). However, this thresholding does not support 3-way crossing areas resulting in holes in the tracking mask and can introduce biases in further analyses such as along tract-profiling and tractometry (Bells et al., 2011; Cousineau et al., 2017). In addition, since FA-based tracking cannot enforce anatomical constraints, it is known to suffer from broken streamlines that terminate prematurely in the white matter (WM). FA-based tracking also suffers from anatomically implausible streamlines that wrongly go through gray matter (GM) and cerebrospinal fluid (CSF) voxels (Girard et al., 2014). This is why nowadays, it is often recommended to use anatomical constraints to ensure streamlines reach GM, subcortical structures, or exit the brainstem, and not terminate in CSF voxels (Smith et al., 2012; Girard et al., 2014). This gave birth to the family of “*anatomically-constrained tractography (ACT)”* algorithms based on more precise masks of WM, GM, and CSF (Smith et al., 2012; Girard et al., 2014; Aydogan and Shi, 2020). More recently, surface-enhanced tractography (SET) (St-Onge et al., 2018, 2021) was proposed to further improve ACT. SET is also based on the same paradigm as ACT but adds another constraint using the cortical surface mesh to initialize tracking and enforce the ending streamline segment orthogonal to the cortex.

Adding a layer of anatomical prior has obvious benefits but also comes with its computational challenges, as an error in this prior often leads to inadequate or suboptimal tractography. Hence, in principle, all algorithms based on ACT or SET require voxel-perfect tissue segmentation in diffusion space to work optimally. Currently, tissue segmentation algorithms such as FSL-fast (Zhang et al., 2001), Atropos (Avants et al., 2009), or Freesurfer (Fischl, 2012) are based on T1- or T2-weighted images and thus, always require a registration step to bring the segmented tissue maps into diffusion space. This registration step is not perfect and is sensitive to preprocessing steps such as brain extraction (Chen et al., 2019). It is also a step that can take multiple hours (from 1 to 10 h), depending on the tool used. Additionally, just like registration algorithms, structural segmentation algorithms, despite spectacular improvements over the past couple of years, are not voxel-perfect wither everywhere in the brain. Segmentation errors often occur in partial volume voxels between tissues (e.g., WM-GM partial volume) and in nuclei extraction. Partial volume effects, nuclei not segmented, or registration errors can thus have a negative impact on tractography algorithms. For example, tractography can be incorrectly allowed to end streamlines in partial volume near the ventricles due to segmentation errors and/or registration inaccuracies in that area. Moreover, the absence of nuclei boundaries allows tracking to go through them without meeting a stopping criterion. Furthermore, the registration issues impact endpoints of streamlines and forbid streamlines to traverse narrow WM corridors such as around the insula, external capsule, and other deep structures. As a result, further analysis such as tractometry can be biased and accumulate undesired errors along streamlines.

**Diffusion MRI segmentation** Diffusion-based segmentation algorithms address registration issues that are not voxel-perfect, which can have a negative impact on tractography results. Moreover, segmenting in diffusion MRI space permits to obtain tissue maps faster in the whole pipeline removing the dependency on T1w preprocessing and T1w registration. For these reasons, diffusion-based segmentation algorithms have started to appear as promising tools in the literature. Recently, Zhang et al. (2021) developed a deep learning algorithm to segment dMRI into WM/GM/CSF classes. The model was trained, validated, and tested on 190 young subjects (under 40 years old) from 5 databases (Glasser et al., 2013; Casey et al., 2018; Tong et al., 2020; Garza-Villarreal et al., 2021). In addition, it required a multi-shell dMRI acquisition to extract kurtosis features (Jensen and Helpern, 2010; Steven et al., 2014), which could be considered a limitation as such a technique cannot work on single-shell clinical dMRI acquisitions. On the other hand, Little and Beaulieu (2021) created an algorithm to segment the GM ribbon based on a combination of fractional anisotropy from DTI and the powdered average DWI from a single-shell diffusion acquisition. Their method was designed to quantify DTI measures under the cortex in a young and healthy cohort of 15 adults. Hence, these two segmentation algorithms were not meant to enhance tractography. Moreover, these two algorithms were developed only on subjects under 40 years of age and could, thus, be misadapted for elderly subjects with larger ventricles, brain atrophy, and white matter hyperintensities. Furthermore, both the Little and Beaulieu (2021) and Zhang et al. (2021) methods did not fully manage deep nuclei, which are very important in tractography to retrieve bundles that connect cortical GM to nuclei such as the optic radiations, among others. Other DWI-based segmentation algorithms permit to extract a 3-class WM/GM/CSF segmentation (Li et al., 2006; Liu et al., 2007; Saygin et al., 2011; Ye et al., 2012; Yap et al., 2015; Zhang et al., 2015; Visser et al., 2016; Battistella et al., 2017; Ciritsis et al., 2018; Nie et al., 2018; Cheng et al., 2020; Wang et al., 2020) but, as the two methods previously presented, they are not adapted to enhance the tractography.

**Contributions of our study** To address the aforementioned issues, we present DORIS: a novel diffusion MRI-based 10 tissue class deep learning segmentation algorithm tailored to improve anatomically-constrained tractography. DORIS is based on a DenseUnet model (Kaku et al., 2019), a convolutional neural network composed of dense blocks in the encoder and decoder path. DORIS is trained and validated on 1,000 subjects from 22 to 90 years old with single and multi-shell acquisitions from 5 databases. In the absence of a “true” ground truth in diffusion space, DORIS uses a silver standard strategy from Freesurfer (Fischl, 2012) output registered onto the DWI. Even if not a perfect ground truth or gold standard, this strategy will be extensively evaluated and discussed later. To train our model, we test several different potential diffusion-based features as input. We test 5 DTI- and HARDI-derived measures as input channels and also test 3 other input channel variants including rotation invariant features (Zucchelli et al., 2020), DTI, and spherical harmonic measures. These 4 input channel variants highlight the possibility of using single and/or multi-shell acquisitions and easily adding DORIS in a dMRI processing pipeline. DORIS predicts a total of 10 tissue class labels: the WM, the GM, the ventricles, the CSF around the brain, and 6 subcortical regions (putamen, pallidum, hippocampus, caudate, amygdala, and thalamus). The goal of DORIS is to segment WM, GM, ventricles, and nuclei voxel maps from diffusion MRI data *only* so that they can be used to perform anatomically-constraint tractography. In summary, this study presents: (i) a large training and validation set covering a wide range of DWI acquisitions and ages, (ii) extensive testing of optimal diffusion measures that drive the DenseUnet algorithm, (iii) a quantitative evaluation of DORIS against Freesurfer and FSL-fast, (iv) speed acceleration compared to well-known segmentation algorithms, and (v) qualitative and quantitative anatomically-constrained tractography benefits.

## 2. Methods

### 2.1. Datasets

Training and validation datasets come from the Alzheimer's Disease Neuroimaging Initiative (ADNI), the Parkinson's Progression Markers Initiative (PPMI), the Human Connectome Project (HCP-1200), and the UK Biobank databases.

Alzheimer's Disease Neuroimaging Initiative was launched in 2003 as a public-private partnership, led by Principal Investigator Michael W. Weiner, MD. The primary goal of ADNI has been to test whether serial magnetic resonance imaging (MRI), positron emission tomography (PET), other biological markers, and clinical and neuropsychological assessment can be combined to measure the progression of mild cognitive impairment (MCI) and early Alzheimer's disease (AD). Data were also obtained from the Parkinson's Progression Markers Initiative (PPMI) database (http://www.ppmi-info.org/access-data-specimens/download-data). HCP-1200 data is described in Glasser et al. (2013) and information about the UK Biobank database can be found at www.ukbiobank.ac.uk. As seen in Table 1, these databases cover a wide range of ages (22–90 years old). A total number of 1,000 subjects were used for training and validation sets, 750 subjects for training and 250 for validation.

**Table 1:** Datasets (training, validation, or testing set), age distribution, number of subjects, *b*-value, number of directions and resolution in mm^3^ isotropic (excepted ADNI) for ADNI, PPMI, HCP, UKBiobank (UKB), and Penthera3T (P3T).

**Database**	**Usage**	**Age range**	**Nb subjects used**	***b*-value(s)**	**Nb dirs**	**Resolution**
ADNI	Train/valid/testing^*^	60–90	250	1,000	41	1.3 x 1.3 x 2.7
PPMI	Train/valid	35–80	250	1,000	64	2
HCP	Train/valid/testing^*^	22–35	250	1,000, 2,000, 3,000	90, 90, 90	1.25
UKB	Train/valid	40–69	250	1,000, 2,000	50, 50	2
P3T	Testing	24–30	72^**^	300, 1,000, 2,000	8, 32, 60	2

* For illustration and example purpose.

** 12 subjects were scanned six times (two sessions with three scans per session).

Testing data comes from the Penthera3T (Paquette et al., 2019) database. Penthera3T is a test-retest database containing scans of 12 subjects from 24 to 30 years old. Each subject was scanned 6 times (two sessions of three scans per session) for a total of 72 acquisitions. We used the 72 acquisition to evaluate DORIS (testing dataset) and be able to evaluate the reproducibility of the segmentation in test-retest. Moreover, some subjects from ADNI and HCP will be used to gauge the performance of DORIS and the impact it has on the tractography.

### 2.2. Data processing

Data processing was mostly done using Nextflow pipelines (Di Tommaso et al., 2017; Kurtzer et al., 2017). Train, validation, and test sets were preprocessed using the TractoFlow pipeline (Theaud et al., 2020) with its default parameters. *B*-values below 1,200 s/mm^2^ were used for DTI metrics while fiber orientation distribution function (fODF) metrics were computed with *b*-values above 700 s/mm^2^. The fODF was generated using a spherical harmonics order of 8, the same fiber response function (Descoteaux, 2008) for all the subjects (15, 4, 4) x 10^−4^ mm^2^/s and with all the shells that required the previously presented requirement. Freesurfer (Fischl, 2012) was used on the raw T1 weighted images. Further details on Freesurfer are provided in Section 2.5.

**Figure 1:**
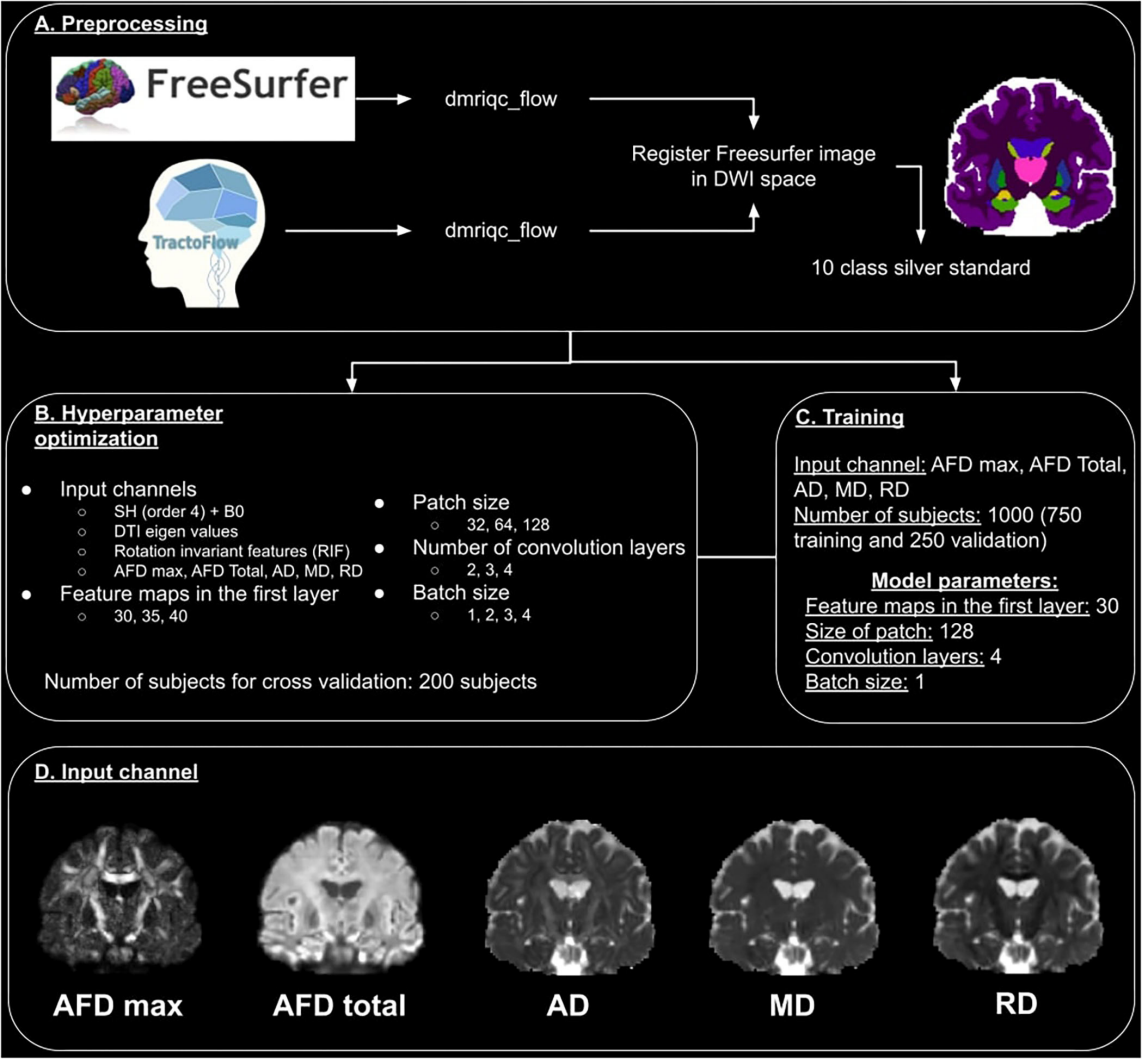
Overview of the DORIS processing pipeline. **(A)** Represents the preprocessing pipeline used for training, validation, and test datasets. **(B)** Illustrates the hyperparameter optimization and input channel selection. **(C)** Describes the training process. **(D)** Illustrates the 5 images used as an input channel.

The data processing pipeline is illustrated in Figure 1A. Data generated by TractoFlow (Theaud et al., 2020) and Freesurfer (Fischl, 2012) were manually verified using a quality control (QC) step with dmriqc flow https://github.com/scilus/dmriqc_flow. Full data processing took 15,000 CPU h, 2,000 GPU h, and 4 Tb of storage. Manual quality control took the authors 50 h of work.

### 2.3. Model

DenseUNet is the deep neural network upon which DORIS is built. This model's architecture was presented by Kaku et al. (2019) and had a similar objective to our study by proposing a segmentation algorithm of brain tissues in several labels based on T1w. Moreover, in this study, they compared the DenseUNet with a classical U-Net that was, in their specific case, less accurate than the DenseUNet. Due to excellent segmentation capabilities and the similarity of Kaku et al. (2019) with our segmentation objective, the DenseUNet was selected to segment diffusion MRI due to its. This model selection choice will be further discussed in the Section 4.

For DORIS, we replaced ReLU with LeakyReLU and, due to memory constraints, image patches were used instead of the full image. In addition, we used exponential logarithmic loss (Wong et al., 2018) developed for unbalanced labels and small structures. This loss is defined as:


(1) $$\begin{array}{r} L_{Exp}=w_{Dice}L_{Dice}+w_{CE}L_{CE}, \end{array}$$

with *w*_*Dice*_ and *w*_*CE*_ being weights for the Dice loss *L*_*Dice*_ and the cross entropy loss *L*_*CE*_. The learning rate was chosen to be 0.0001. Moreover, we used data augmentation (rotation, scaling, shearing, and axis flip) on the training set (value ranges for rotation, scaling and shearing are available in Supplementary Materials). Training of the Dense-UNet went on for 163 epochs before it was stopped by an early stopping criterion.

### 2.4. Hyperparameter optimization

We optimized 5 hyperparameters listed in Figure 1B. This includes four different input channels i.e.,:

1. DWI (*b* = 1,000 s/mm^2^) fitted with spherical harmonics (SH) of order 4 with b0 concatenated (to add the non-diffusion T2w contrast). This image is a simplified representation of the DWI.
2. The 6 eigenvalues of the tensor matrix from DTI fit. The eigenvalues are the rotation invariant features of the diffusion tensor.
3. The rotation invariant features (from *b* = 1,000 s/mm^2^ DWI) (Zucchelli et al., 2020) of the spherical harmonics. These are the 4th-order HARDI shape representation equivalent of the DTI eigenvalues.
4. Four DTI- and HARDI-derived measures include maximum and total apparent fiber density (AFD max and AFD Total), the DTI axial diffusivity (AD), mean diffusivity (MD), and radial diffusivity (RD). As pointed out by Chamberland et al. (2019), these 4 diffusion measures are good representative features that maximize the variance of the dMRI data using principal component analyses (PCA).

These images were selected because of the straightforwardness of computing them with public open-access software and because they can be extracted from any single-shell DWI acquisition, which makes them more suitable for future translation to a wide range of applications.

We also optimized the number of feature maps in the first layer (30, 35, and 40), the patch size (32 x 32 x 32, 64 x 64 x 64, and 128 x 128 x 128), the number of convolution layers (2, 3, and 4), and the batch size (1, 2, 3, and 4). These hyperparameters were optimized with data of 200 subjects from the dataset previously described.

The hyperparameter search revealed that the best input channel to use is the 4D image made of AFD max, AFD Total, AD, MD, and RD images (Chamberland et al., 2019). A representative illustration of these 5 images is shown in Figure 1D. The best number of feature maps in the first layer is 30, the best patch size is 128, the best number of convolution layers is 4 and the best batch size is 1. The summary of this optimization is shown in Figure 1C.

### 2.5. Silver standard

**Figure 2:**
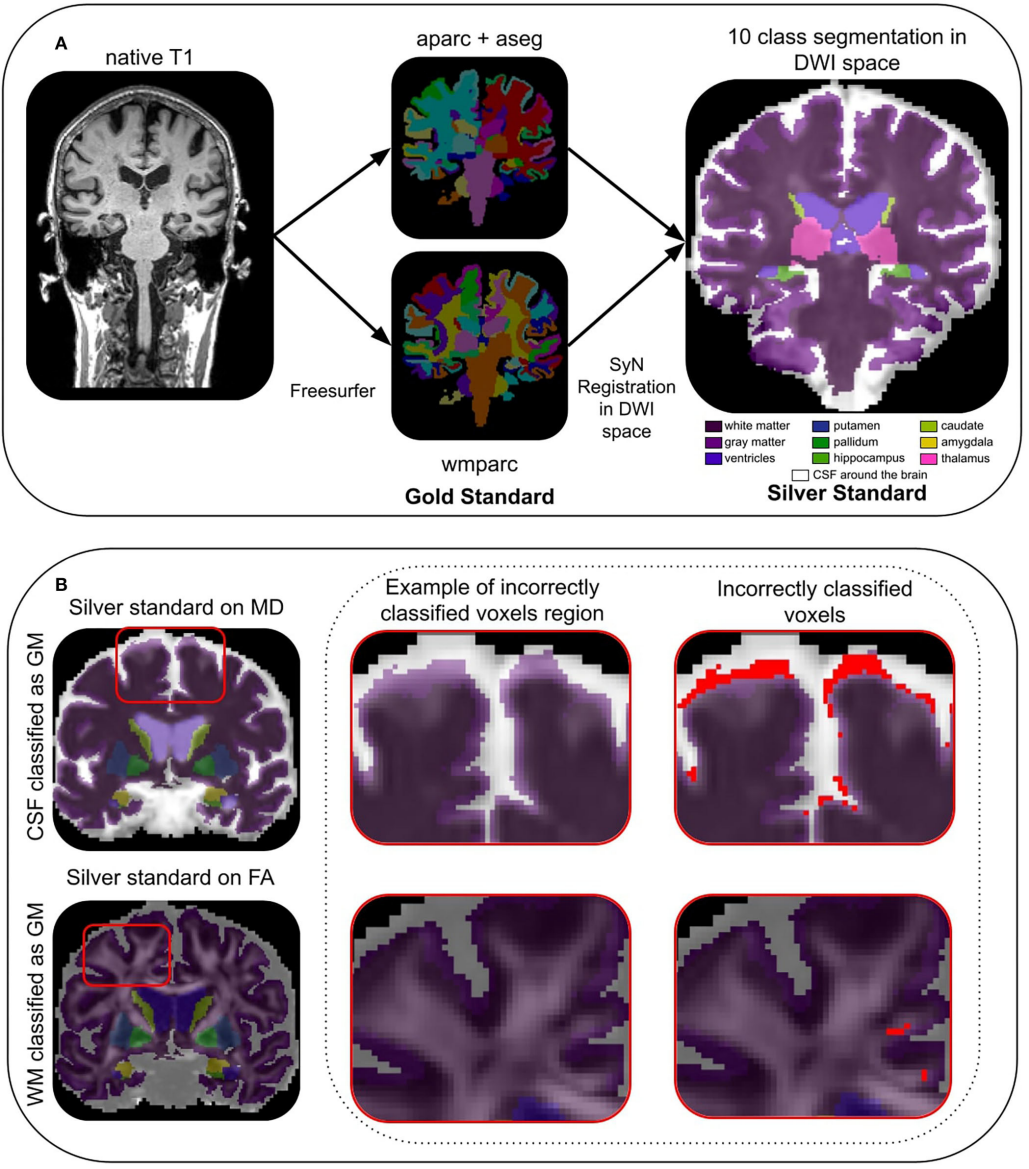
In **(A)**, the process of generating the silver standard in DWI space from Freesurfer computed in the native T1 space. In **(B)**, an example of CSF (row 1) and WM (row 2) incorrectly classified as GM (in red), in the last column.

Since the notion of “true” ground truth (or gold standard) is unavailable in diffusion space, we used Freesurfer (Fischl, 2012) to create our “reference maps” in diffusion space, or what we prefer to call our **silver standard**. As illustrated in Figure 2A, the native T1-weighted images were first processed by Freesurfer 6.0 to generate wmparc and aparc+aseg images, which were then registered in diffusion space using an ANTs registration operation (the exact command is available in the Supplementary Materials) (Avants et al., 2009). The registration used the nearest neighbor interpolation and an affine + non-linear SyN warping transformations from the Tractoflow output. Next, we concatenated Freesurfer labels from these registered wmparc and aparc+aseg images to create our **silver standard** reference maps with the following 10 labels: (1) white matter, (2) gray matter, (3) ventricles, (4) putamen, (5) pallidum, (6) hippocampus, (7) caudate, (8) amygdala, (9) thalamus, and (10) CSF around the brain, as seen in Figure 2.

#### 2.5.1. Incorrectly classified voxels

Due to the registration step, our silver standard suffers from issues that we previously described, i.e., some voxels are wrongly aligned with the dMRI data, which leads to **incorrectly classified voxels**. This is expected considering the nature of our silver standard construction methodology. Some of these incorrectly classified voxels can be automatically detected using measures in diffusion space such as MD and FA from DTI maps. Based on a theoretical CSF mean diffusivity of 3 × 10^−3^*mm*^2^/*s* reported in the literature (Koo et al., 2009; Pasternak et al., 2009; Zhang et al., 2012), a “safe” CSF mask (Dumont et al., 2019) containing the ventricles and the part of the constrained CSF between the skull and the brain is extracted. To extract this “safe” CSF mask, MD voxels higher than 2 × 10^−3^*mm*^2^/*s* (Groen et al., 2011) were selected. To quantify the number of CSF voxels classified as GM voxels, we intersect the “safe” CSF mask with the GM region of DORIS as well as that of the silver standard. As shown in Figure 2B, the number of voxels in the two intersections corresponds to the number of CSF voxels incorrectly classified as GM. For the “safe” WM mask (Dumont et al., 2019), we considered voxels with a FA value above 0.3 as WM (Chamberland et al., 2014). Then, we intersect the GM label with the “safe” WM mask. The number of voxels in the two intersections corresponds to the number of WM voxels incorrectly classified as GM. Finally, note that the CSF around the brain label will only be used to compute the incorrectly classified voxels and not used in the other analysis. Indeed, CSF around the brain is not useful for tractography purposes.

Incorrectly classified voxels will permit us to address the limitation of our silver standard and explore solutions, with DORIS in native diffusion space, that could potentially have fewer incorrectly classified voxels than in the silver standard itself. The goal of DORIS is to generate fewer incorrectly classified voxels than the silver standard due to the large number of subjects in the training set and to diffusion measures inputted into the model. This issue will be extensively discussed in the Section 4.

### 2.6. Evaluation

The DORIS evaluation consists of 7 steps:

1. Qualitative results of DORIS on ADNI and HCP subjects,
2. A computation time comparison between DORIS and state-of-the-art algorithms (Fastsurfer; Henschel et al., 2020, FSL-fast; Zhang et al., 2001, and Freesurfer; Fischl, 2012),
3. A comparison between a manual segmentation, DORIS, and the silver standard in a small region-of-interest (ROI),
4. The number of incorrectly classified voxels generated by DORIS compared to the silver standard,
5. A comparison between the volume of labels from DORIS and the silver standard to ensure the reproducibility in test-retest,
6. A Dice score (Dice, 1945) between DORIS and the silver standard,
7. Quantitative and qualitative analyses about the impact of DORIS on tractography.

#### 2.6.1. Evaluation using a manual segmentation

As introduced previously, our silver standard is imperfect. Thus, to compare DORIS with the silver standard, an ROI located near the insula (illustrated in Figure 6) was manually segmented by an expert. We targeted that region due to its complexity of being segmented. Indeed, this area is composed of small white matter corridors between the insula, the putamen, the thalamus, and the caudate nuclei. To facilitate the manual segmentation process, nuclei were not separated from the GM cortex, i.e., they are both considered the same label for this evaluation experiment. To have a better analysis of DORIS, the manual segmentation will be used as a reference. For DORIS and the silver standard, the same labels were extracted from the ROI to make a qualitative analysis. Finally, quantitative analysis will be done by computing the Dice score between DORIS and the manual segmentation; and between the silver standard and the manual segmentation.

#### 2.6.2. Evaluation using Penthera3T

**Incorrectly classified voxel** To determine if one of the segmentation generates significantly more outliers than the other, a two-sided *t*-test was also performed between incorrectly classified voxels generated by DORIS and the silver standard. As for the volume test, the significance level was fixed at p < 0.001.

**Volume** For DORIS and the silver standard, we compute the volume in mm^3^ of each of the 9 labels (excluding the CSF around the brain). To validate if the volume is significantly different, a related two-sided *t*-test is performed between sessions for DORIS and the silver standard. The volume is determined as significantly different in the case where p < 0.001 (using scipy). Moreover, the volume for each label is averaged across the full dataset to obtain a global mean volume for each label.

**Dice** For each label and each acquisition, we compute the Dice score (Dice, 1945) between DORIS and our silver standard. Then, we average the Dice scores of each subject to obtain one average Dice score per label across all the testing datasets. We also compute 2 additional WM map DWI based as a comparison based on classical FA threshold higher than 0.15 and WM signal fraction from multi-shell multi-tissue (MSMT) fODF (Jeurissen et al., 2014) higher than 0.1.

#### 2.6.3. Impact of DORIS on tractography

To evaluate the impact of DORIS on tractography, we performed a particle filtering tractography (PFT) (Girard et al., 2014), which is part of the ACT family of algorithms (Smith et al., 2012). The PFT algorithm is known to be more restricted by probabilistic tissue maps. Acquisitions used for this qualitative analysis come from ADNI and Penthera3T database to cover a large age range of dMRI quality and anatomical difficulty (less WM in ADNI, enlarged ventricles, thinner cortex, presence of white matter hyperintensities). To compare our DORIS-based tractograms, we also compute a standard tractogram using FSL-fast maps, as done in Tractoflow (Theaud et al., 2020). Tracking parameters were the same for both techniques: a probabilistic tractography using a step size of 0.5mm and a maximal angle between two steps of 20 degrees. The fODF used in both tracking was computed using a spherical harmonic order of 8. We launched 5 seeds per voxel from the WM mask (obtained from DORIS or FSL-fast).

To extract white matter bundles, we ran RecobundleX (RBx) (Garyfallidis et al., 2015; Rheault, 2020) on both DORIS and FSL-Fast Tractoflow tractograms. From RBx, 6 bundles were used for quantitative and qualitative analyses: (i) Superior longitudinal fasciculus (SLF), (ii) whole corpus callosum (CC), (iii) Inferior fronto-occipital fasciculus (IFOF), (iv) fornix (FX), (v) anterior, and (vi) posterior commissure (AC/PC). Based on Maier-Hein et al. (2017), we considered the SLF and CC as “easy to track” bundles, the IFOF as “hard to track” bundles, and the FX and AC/PC as “very hard to track” bundles. For these 6 bundles, the number of streamlines and the mean bundle length are reported. Finally, the number of streamlines per bundle is divided by the total number of streamlines and expressed as a percentage (*ratio* = (*number of streamlines in the bundle* / *number of streamlines in the tractogram*) × *100*).

## 3. Results

### 3.1. DORIS segmentation on ADNI and HCP

**Figure 3:**
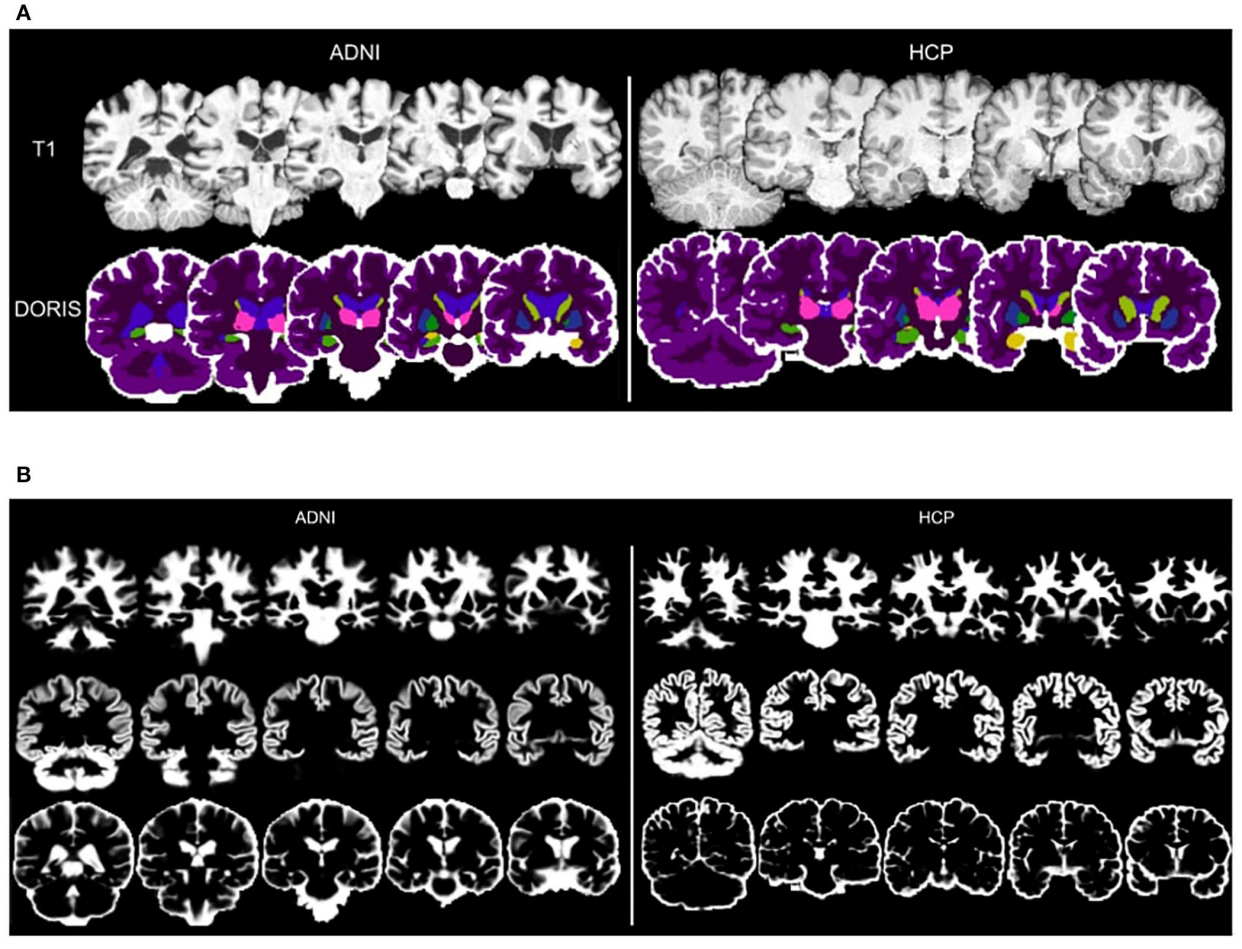
Binary and probability segmentation maps obtained from DORIS on ADNI and HCP subject. **(A)** DORIS binary segmentation on a ADNI and HCP subject. T1 image illustrate differences between an aging and young brain. **(B)** DORIS probability segmentation. of WM, GM and CSF (combining ventricules and CSF around the brain) on a ADNI and HCP subject. The mosaic containing the nuclei is available in Supplementary Materials.

**Figure 4:**
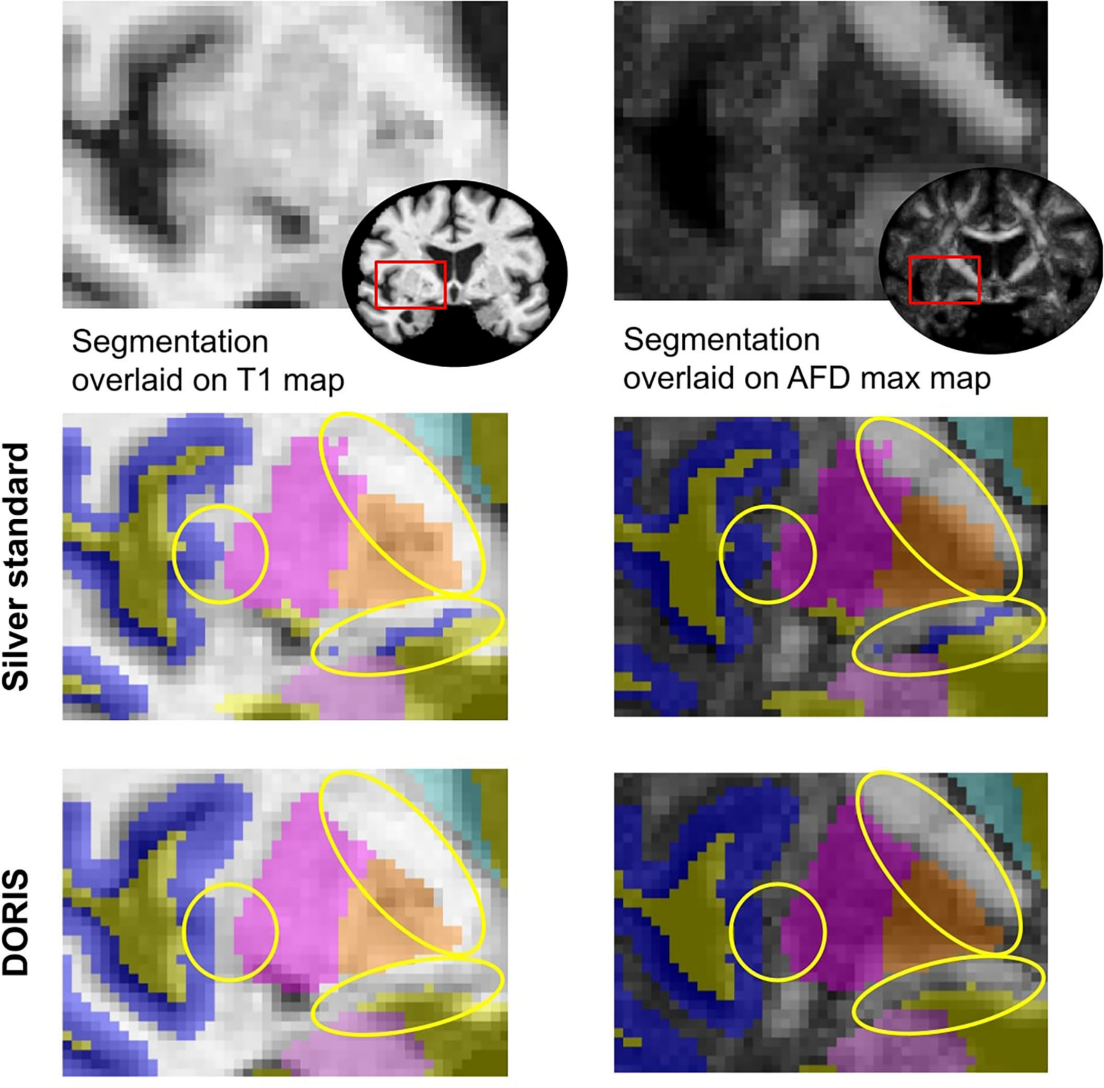
Silver standard and DORIS segmentation near the insula overlaid on the T1 and AFD max map. Yellow circles show where DORIS has a better segmentation than the silver standard.

Figure 3A shows a qualitative example of DORIS (bottom row) performance on ADNI and HCP subjects. Figure 3B shows, for the same subjects, the probabilistic maps obtained by DORIS. For the ADNI subject, DORIS correctly classified the WM even with the presence of aging lesions (white matter hyperintensities). Moreover, DORIS separates the GM cortex from the nuclei (see Supplementary Materials for nuclei figure). As shown in Figure 4, DORIS is better than the silver standard to segment the nuclei and tiny white matter corridors.

**Figure 5:**
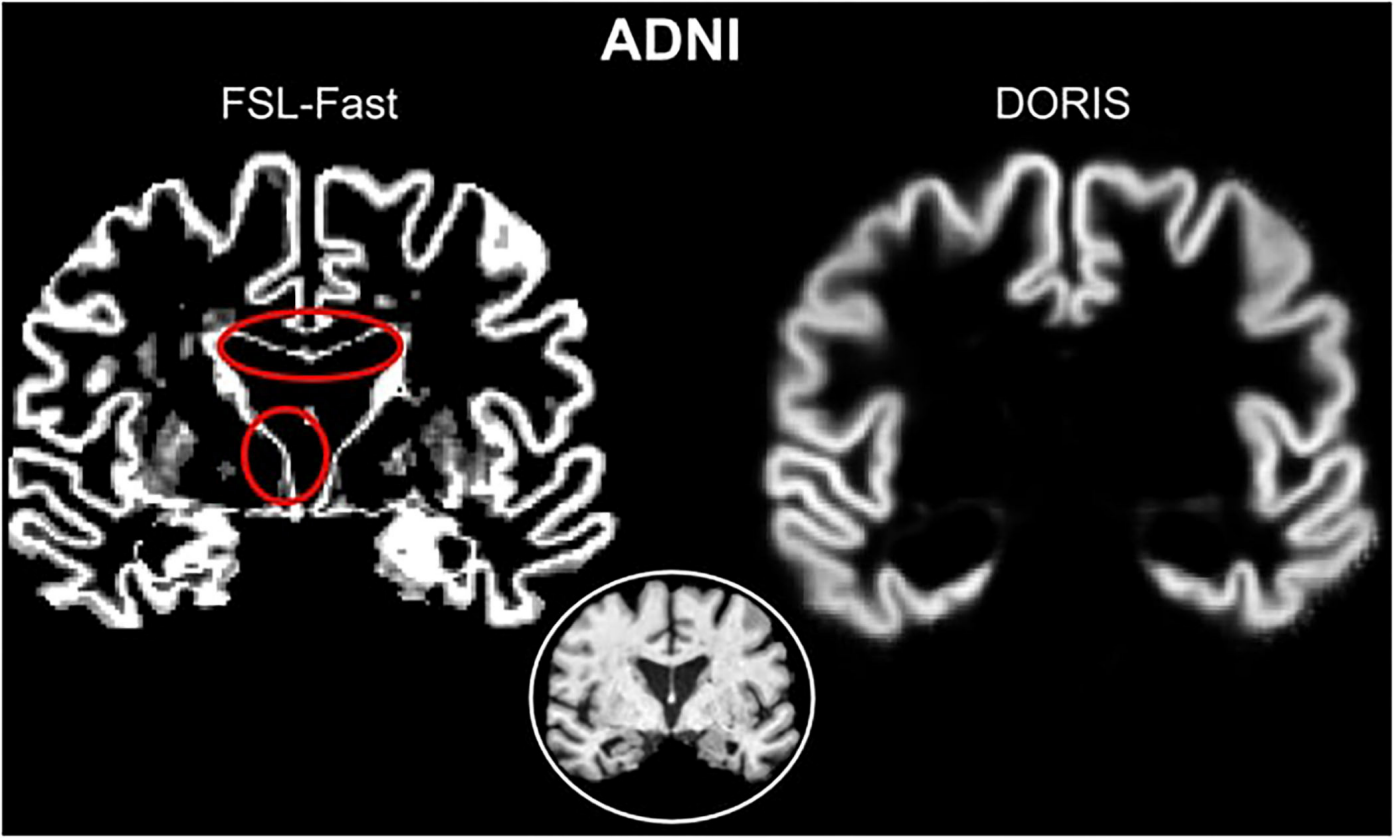
Red circles show WM-CSF partial volume incorrectly classified as GM in FSL-Fast. However, DORIS does not classify these partial volume voxels as GM.

Then, as illustrated in Figure 5 with an ADNI subject, DORIS does not classify WM-CSF partial volume (around the ventricles) as GM. This misclassification of WM-CSF partial volume is visible in FSL-Fast segmentation. To easily compare DORIS to FSL-Fast and the silver standard, figures with probabilistic maps obtained by FSL-Fast and silver standard segmentation on these two subjects are available in Supplementary Materials.

### 3.2. Computation time

We evaluated the computation time of DORIS compared to three well-known segmentation algorithms based on T1. Table 2 shows the computation time for each algorithm. DORIS has a similar computation time to FastSurfer (48 and 42 s, respectively), it is faster than FSL-fast (approximately 2 min) and orders of magnitude faster than the *recon-all* command from Freesurfer (10 h). It is important to note that even if some algorithms (FastSurfer or FSL-Fast) have a computation time comparable to DORIS, they need an extra 45 min to bring their segmentation map into the diffusion space.

**Table 2:** Computation time per subject for DORIS, FastSurfer, FSL-Fast, and FreeSurfer.

**Algorithm**	**Computation time**	**SyN registration time**
DORIS	48 s	N/A
FastSurfer	42 s	45 m
FSL-Fast	2 m: 12 s	45 m
Freesurfer	10 h	45 m

In the last column, SyN registration computation time are reported to bring the T1 image into DWI space is described.

### 3.3. Manual segmentation

**Figure 6:**
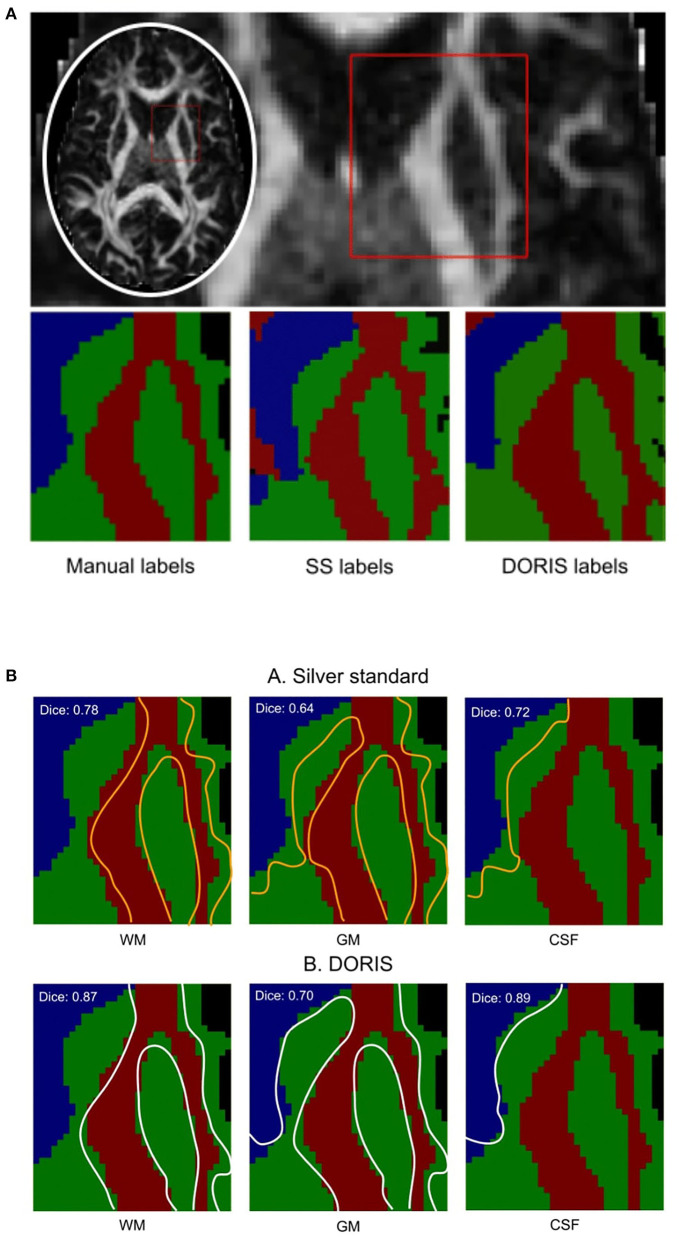
Manual segmentation qualitative and quantitative analyses. Based on the Dice score and segmentation alignment, DORIS is more in agreement with the manual segmentation than the silver standard. **(A)** Region of interest outlined in red square for qualitative and quantitative analyses of the three segmentation results (Manual, Silver Standard (SS) and DORIS). **(B)** Comparison between manual segmentation and silver standard **(A)**, and DORIS **(B)**. Tissue edges of the silver standard (orange) and DORIS (white) are overlaid on the manual segmentation for qualitative assessment of the segmentation quality.

Figure 6B shows the manual outline of the silver standard (A) and DORIS (B) overlaid on top of the manual segmentation maps. The outlines of DORIS are in better agreement with the manual segmentation than the silver standard, especially along the GM and CSF edges. The mean Dice score of DORIS with respect to the manual segmentation is 0.82, which reveals a good agreement. For the WM, GM, and CSF, DORIS Dice scores are respectively 0.87, 0.70, and 0.89. As for the silver standard, its Dice scores are lower with 0.71 for the mean Dice score and 0.78, 0.64, and 0.72 for the WM, GM, and CSF. Overall, this experience shows a Dice score increase, between the silver standard and DORIS, of 11.5% for the WM, 9.4% for the GM, and 23.6% for the CSF.

### 3.4. Penthera3T

#### 3.4.1. Incorrectly classified voxels

**Figure 7:**
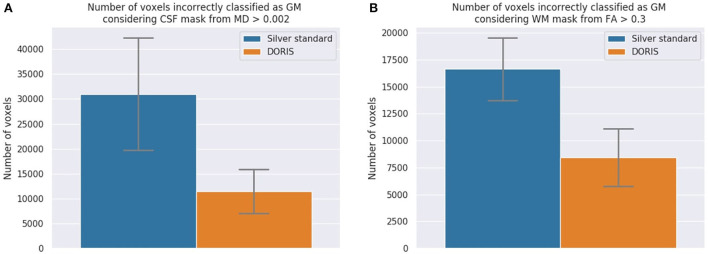
The number of incorrectly classified WM and CSF voxels. DORIS predicts significantly fewer CSF and WM voxels incorrectly classified as GM than the silver standard. **(A)** CSF voxels incorrectly classified as GM. Significance level of *p* < 0.001 is reached and indicates significantly more incorrectly classified voxels in the silver standard than DORIS. **(B)** WM voxels incorrectly classified as GM. Significance level of *p* < 0.001 is reached and indicates significantly more incorrectly classified voxels in the silver standard than DORIS.

**CSF voxels classified as GM** First, we present the number of CSF voxels incorrectly classified as GM. As shown in Figure 7A, while DORIS generates 11,459 incorrectly classified voxels, the silver standard generates 3 times as many incorrectly classified voxels: 30,999. This corresponds to a 171% significant increase of incorrectly classified voxels for the silver standard compared to DORIS (related *t*-tests *p* = 1.13 × 10^−31^).

**WM voxels classified as GM** Figure 7B shows the number of WM voxels incorrectly classified as GM. DORIS gets 8,422 incorrectly classified voxels against twice as many for the silver standard. This corresponds to a significant increase of 98% (related *t*-tests *p* = 2.12 × 10^−37^).

#### 3.4.2. Volume

**Figure 8:**
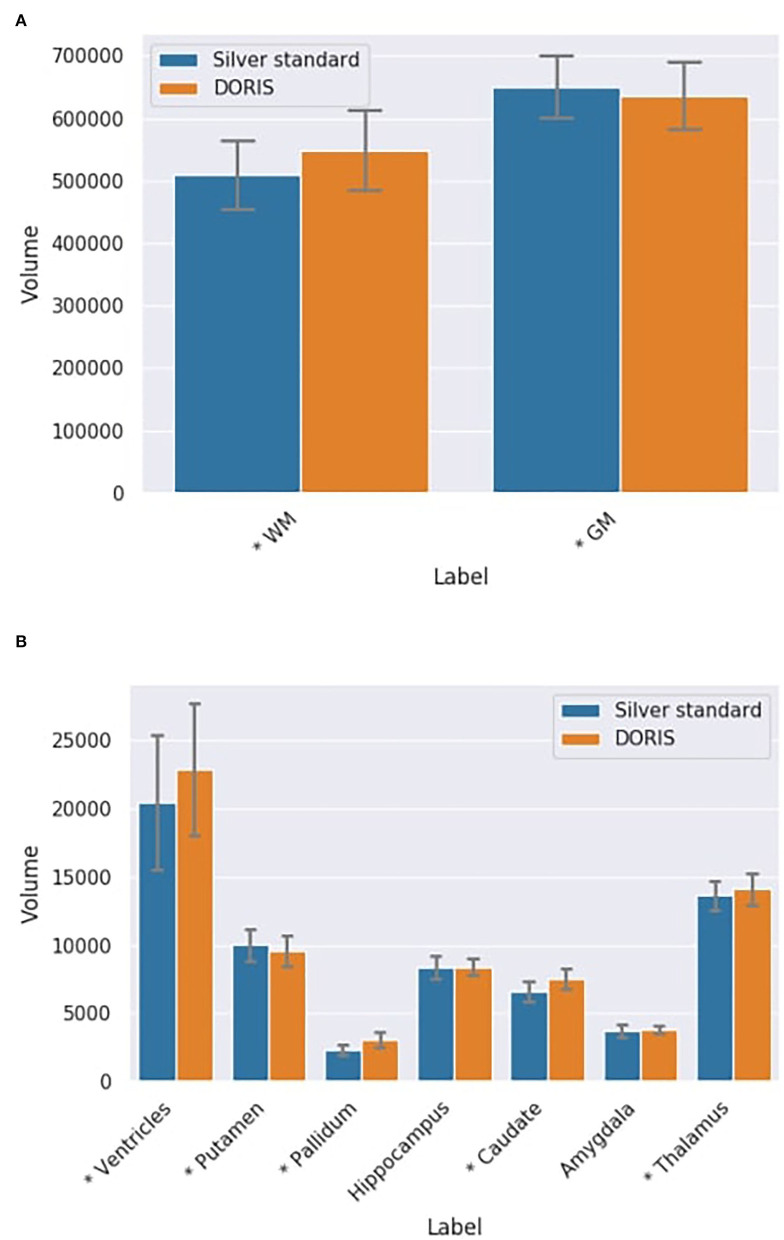
Label volumes in mm^3^ for DORIS and the silver standard. DORIS and silver standard have label volumes significantly different except for the hippocampus and the amygdala. **(A)** WM and GM volumes in mm^3^ (*p < 0.001) for DORIS and the silver standard. **(B)** Small label volumes in mm^3^ (*p < 0.001) for DORIS and the silver standard.

At first, we compare the volume of each region between DORIS and the silver standard using the testing dataset, ignoring the test-retest for now. For the WM and GM regions, Figure 8A shows a certain volume difference between DORIS and the silver standard. The average WM volume for DORIS is 5,49,175 mm^3^ (std: 65,178 mm^3^) against 5,09,096 mm^3^ (std: 55,475 mm^3^) for the silver standard; an increase of 7.9%. As for the GM, DORIS gets and average volume of 6,36,705 mm^3^ (std: 53,742 mm^3^) against 6,50,521 mm^3^ (std: 50,136 mm^3^) for the silver standard volume an increase of 2.2%. The WM and GM volume are significantly different between DORIS and the silver standard. Related *t*-test results for WM and GM volumes between DORIS and the silver standard are available in Supplementary Materials.

Figure 8B shows volumes for the smallest regions (ventricles, putamen, pallidum, hippocampus, caudate amygdala, and thalamus labels) produced by DORIS and the silver standard. The percentage of volume differences varied from 0.5% for the hippocampus to 33.5% for the pallidum (a volume table is available in the Supplementary Materials). Except for the hippocampus and amygdala, related *t*-tests revealed significantly different volumes in all the smallest labels between DORIS and the silver standard. Related *t*-test scores are available in Supplementary Materials.

**Figure 9:**
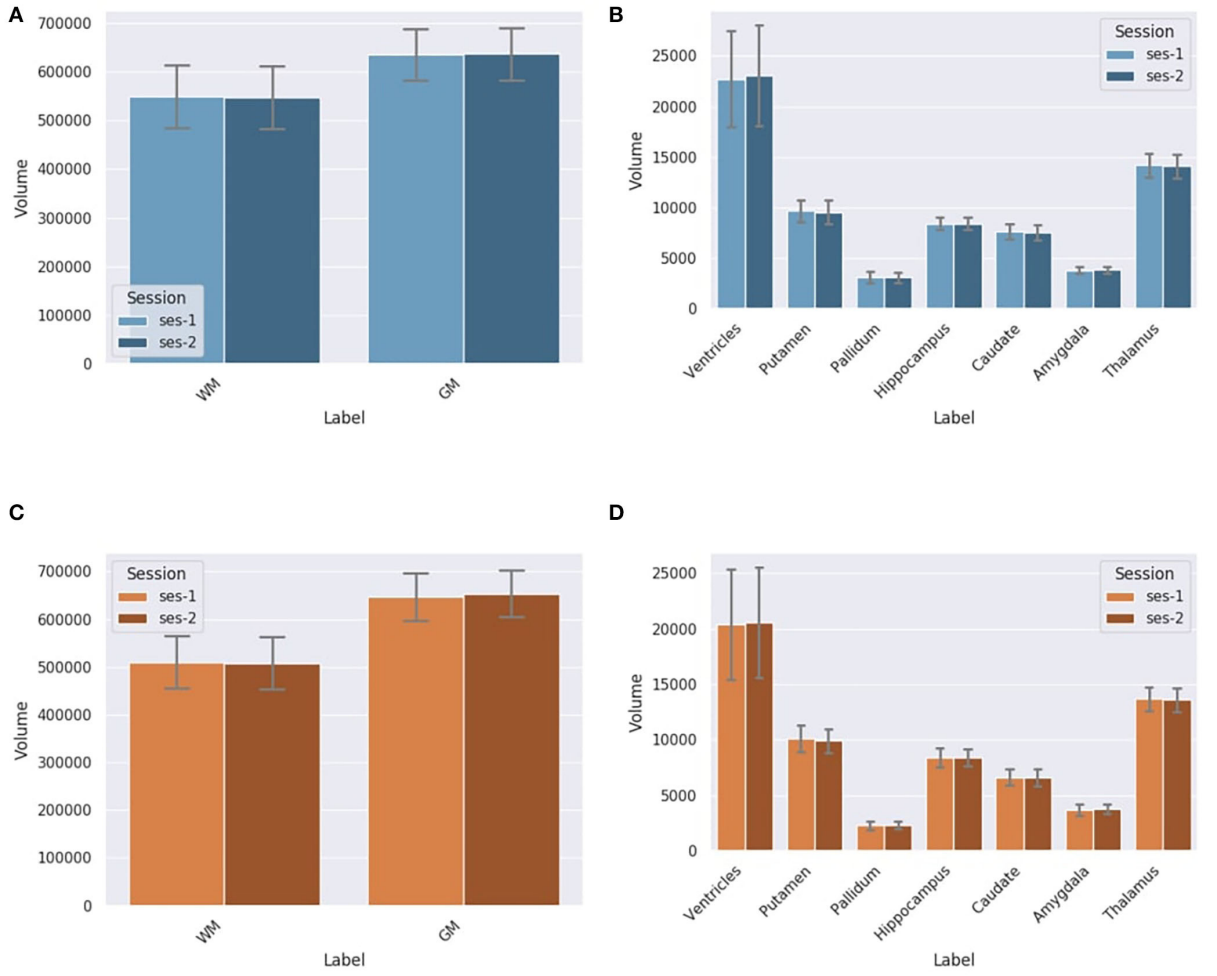
Label volumes in mm^3^ for DORIS and the silver standard (SS) in test-retest for session 1 and session 2. Label volumes in test-retest are not significantly different for DORIS and the silver standard segmentation. **(A)** WM and GM volumes in mm^3^ for DORIS in test-retest for session 1 and session 2. **(B)** Small label volumes in mm^3^ for DORIS in test-retest for session 1 and session 2. **(C)** WM and GM volumes in mm^3^ for the SS in test-retest for session 1 and session 2. **(D)** Small label volumes in mm^3^ for the SS in test-retest for session 1 and session 2.

**DORIS and Silver Standard in test-retest dataset** As seen in Figures 9A,B, there is no statistically significant variation in the DORIS computed volumes in test-retest between the two sessions across all subjects (smallest *p*-value across labels: *p* = 0.107). As seen in Figures 9C,D, similarly to DORIS, no statistically significant differences are observed for the silver standard volumes (smallest *p*-value across labels: *p* = 0.06). Full related *t*-test results for DORIS and the silver standard are available in Supplementary Materials.

#### 3.4.3. Dice

**Figure 10:**
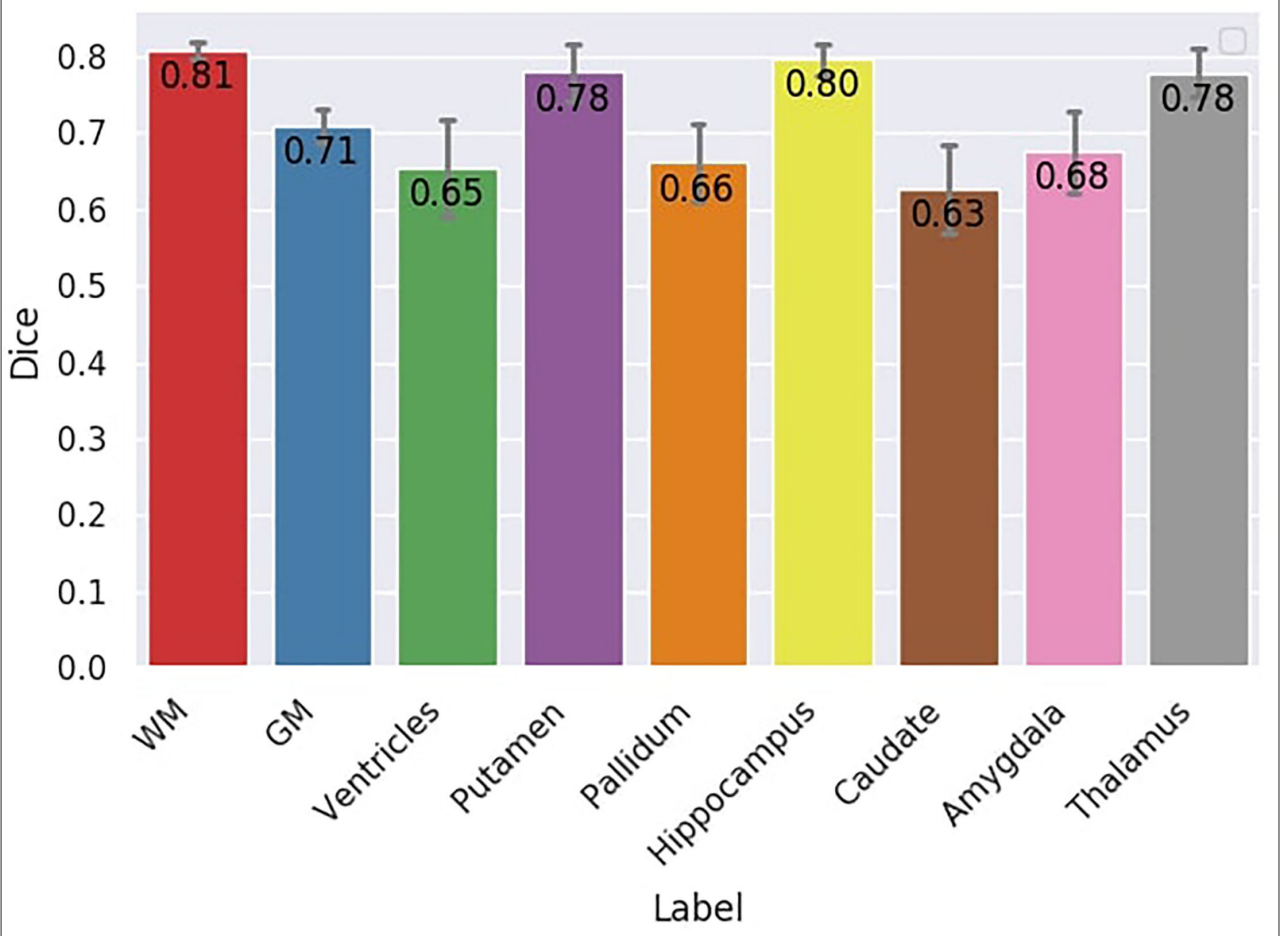
Dice score between DORIS and the silver standard. The Dice score between DORIS and the silver standard varied between 0.63 and 0.81.

Dice scores for each region are illustrated in Figure 10. The mean Dice score across all the regions is 0.72. The lower Dice score is 0.63 for the caudate nuclei and the highest is 0.81 for the WM. FA-based WM had a Dice score of 0.55 compared to the silver standard WM mask, whereas multi-shell multi-tissue based WM had a better Dice score (0.58) than the silver standard. These two methods serve as a reference for the DORIS WM Dice performance. Overall, FA-based thresholding and multi-shell multi-tissue spherical deconvolution from MRtrix (Supplementary Materials) give a generally lower Dice score than DORIS with the silver standard in the whole WM.

### 3.5. Impact of DORIS on tractography

**Figure 11:**
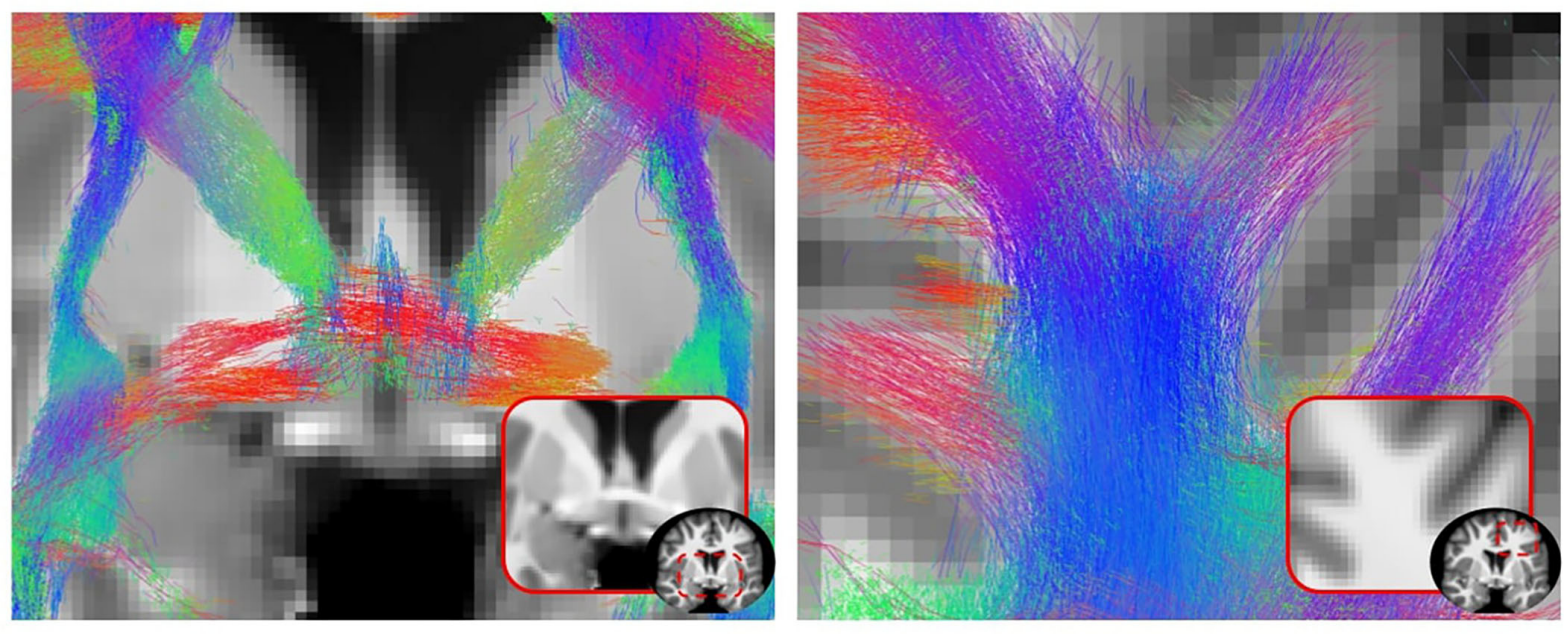
2D view of a particle filtering tractography (PFT) performed using DORIS segmentation on a Penthera3T subject. PFT DORIS-based enables to not go through the nuclei with a good gyrus coverage.

**Figure 12:**
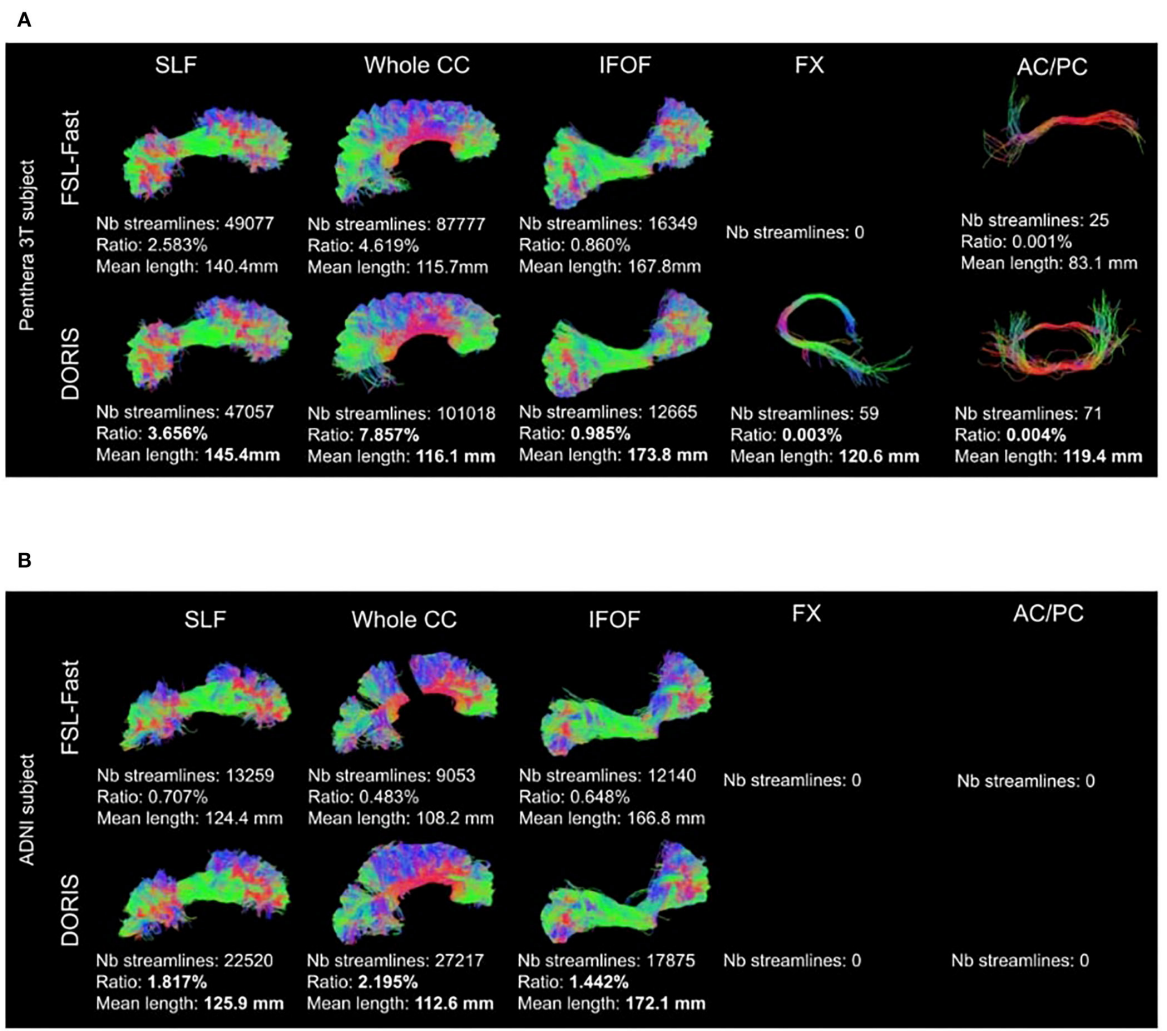
Bundles extracted from **(A)** Penthera3T and **(B)** ADNI subjects. For each bundle, we reported the number of streamlines, the ratio, and the mean length of the bundle. For both Penthera3T and ADNI subjects, DORIS based bundles have a better ratio and mean length. For Penthera 3T, DORIS based tractogram reconstructe the Fornux and the commisures correctly. For the ADNI subject, DORIS based tractogram was not impacted by the presence of aging lesions. **(A)** Superior longitudinal fasciculus (SLF), whole corpus callosum (CC), inferior longitudinal occipital fasciculus (IFOF), Fornix (FX), anterior and posterior commissure (AC/PC) extracted from a Penthera3T subject tractogram based on FSL-Fast (top row) and DORIS (bottom row). **(B)** Superior longitudinal fasciculus (SLF), whole corpus callosum (CC), inferior longitudinal occipital fasciculus (IFOF), Fornix (FX), anterior and posterior commissure (AC/PC) extracted from an ADNI subject tractogram based on FSL-Fast (top row) and DORIS (bottom row).

**Figure 13:**
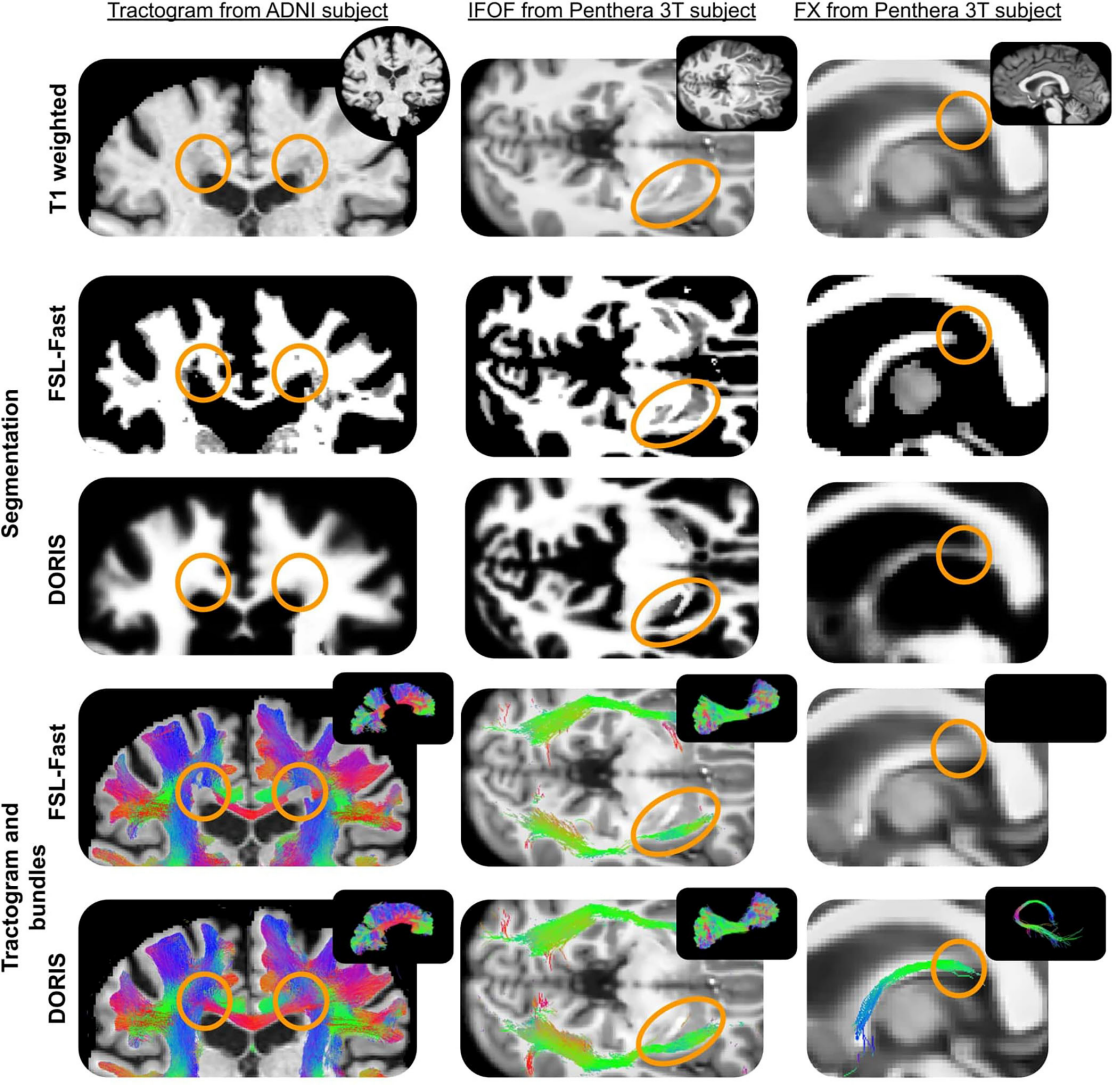
ADNI subject's tractogram and Penthera 3T subject's IFOF and FX are intersected on the T1 map. Orange circles show where DORIS is better than FSL-Fast. In the ADNI subject, the FSL-Fast based tractogram is impacted by the presence of WM lesions whereas the DORIS based tractogram is not impacted. For the Penthera3T subject, DORIS based IFOF do not go in the nuclei and the Fornix bundle is reconstructed.

Figure 11 shows qualitative results of PFT tracking using DORIS-based segmentation maps. These qualitative results underline that the DORIS-based tractogram does not go through the nuclei and enables the exploration of the WM under the gyri, a well-documented hard-to-track region (Maier-Hein et al., 2017; Mandonnet et al., 2018; St-Onge et al., 2018, 2021). Figure 12 also shows the 6 bundles (SLF, whole CC, IFOF, FX, and AC/PC) extracted from Penthera and ADNI subjects. The 6 bundles were retrieved in the Penthera3T subject. However, the FX and the AC/PC were not retrieved in the ADNI subject, which is not surprising due to the brain atrophy and thinning of WM corridors. For the Penthera3T subject (Figures 12A, 13), the FSL-Fast-based tractogram has no streamline in the FX and got only a fraction of the AC/PC. On the other hand, the DORIS-based tractogram contains all 6 bundles. As for the number of streamlines, while the FSL-fast Tractoflow tractogram has slightly more streamlines in the SLF and IFOF, the DORIS-based CC, FX, and AD/PC are in much better shape. For the ratio and the mean length, DORIS outperforms FSL-Fast for the 6 bundles. As for the ADNI subject (Figure 12B), the number of streamlines, the ratio, and the mean bundle length are higher for the 3 bundles (SLF, whole CC, and IFOF) with the DORIS segmentation map. Figure 13 shows, in the ADNI subject, that parts of the whole CC are not extracted due to the presence of WM aging lesions.

## 4. Discussion

DORIS presents a good computation time and outperforms some of the state-of-the-art algorithms doing the segmentation at the same time as FastSurfer, in less than 1 min per subject. Moreover, DORIS has a good accuracy compared to a manual segmentation and exhibits good reproducibility performances in test-retest experiments. DORIS also has fewer incorrectly classified voxels than our silver standard extracted from Freesurfer registered into diffusion space. Finally, empirical results show that DORIS is a good candidate to improve anatomically constrained tractography. With preliminary tractography results, DORIS seems to produce improved anatomically constrained tractograms and, thus, permits the extraction of hard-to-track bundles in tiny corridors like the fornix and the anterior and posterior commissures.

### 4.1. DORIS and the silver standard

Dice scores from the *Results* section show that DORIS performs well and is more accurate than the silver standard compared to the manual segmentation. In addition, manual segmentation analyses show a good overall performance of DORIS, with Dice scores between 0.70 and 0.89. This confirms that the registration step introduces errors that affect the silver standard (as shown in Section 3.2).

With DORIS, the number of incorrectly classified voxels is significantly less than the silver standard. In addition, region volumes across the full testing dataset varied between DORIS and the silver standard. DORIS produces a larger WM volume than Freesurfer. This volume difference is an advantage for tractography algorithms that have access to more voxels to traverse in hard-to-track regions. Even if volumes of tissue labels are different between the silver standard and DORIS, DORIS can be considered more accurate than the silver standard due to its fewer incorrectly classified voxels. Moreover, as shown in Figure 4, the nuclei shapes were better with DORIS than with our silver standard. This better definition helped the tractography reconstruction of some WM bundles, such as the IFOF, that go near these nuclei.

Overall, results suggest that DORIS outperforms the silver standard. This can be explained by the location of silver standard errors that are different between subjects. Silver standard errors are not the same among the subjects even if it is the same region in the brain (illustrated in Supplementary Materials). Hence, given our large training set of 750 subjects, we hypothesize that DORIS was, therefore, able to generalize and make fewer segmentation errors than the silver standard.

### 4.2. Computation time

As shown in Table 2, the registration step adds 45 min of processing time for the state-of-the-art algorithms, a burden that DORIS does not suffer from. However, in a tractography context, the T1 weighted image must also be preprocessed (e.g., denoised, resampled) and then registered in diffusion space (Theaud et al., 2020). Adding other T1 preprocessing steps adds another 1 or 2 h of computation time, depending on the software used. Using DORIS, these 1–2 h of the processing could be optional if the goal is to obtain a whole brain tractogram. However, T1 processing and registration in DWI space remain useful for qualitative or volume analyses.

### 4.3. Limitations

One major limitation of DORIS is the silver standard used for training and validation. As mentioned in the introduction, a proper ground truth in diffusion space does not exist in the community. Even if manual segmentation has proven useful to validate the DORIS segmentation, it is impossible to do this on an entire brain and multiple subjects. This would require countless hours of one or more neuroanatomists who would have to work on 2 mm resolution images switching between *b* = 0 or FA or T1 images (or others) to perform the manual segmentation. Such a gold standard is, thus, unlikely to ever exist. On the other hand, DWI-based segmentation algorithms do exist but none of them have been developed to enhance tractography and all of them produce a maximum of 3 tissue classes (WM, GM, and CSF) (Li et al., 2006; Liu et al., 2007; Yap et al., 2015; Zhang et al., 2015, 2021; Visser et al., 2016; Ciritsis et al., 2018; Nie et al., 2018; Cheng et al., 2020; Wang et al., 2020; Little and Beaulieu, 2021). In the MRtrix3 software (Tournier et al., 2019), WM, GM, and CSF masks can be extracted from signal fractions obtained from multi-shell multi-tissue fODF (Jeurissen et al., 2014). However, as demonstrated in the results, this method is limited and has not been evaluated on tractography yet. Also, WM, GM, and CSF masks can be extracted with a single-shell 3-tissue fODF version developed by Dhollander and Connelly (2016) called the SS3T-CSD method. However, SS3T-CSD signal fraction maps have not been confronted with ACT. Jeurissen and Szczepankiewicz (2021) and Karan et al. (2021) recently showed that having a tensor-value DWI using linear and spherical encoding helps WM, GM, and CSF signal fraction estimation. This method requires a very specific multi-dimensional b-tensor encoding acquisition scheme and is not easily applicable to actual well-known databases such as HCP, ADNI, and UKBiobank. For all the above reasons, we chose to use our silver standard, based on T1 segmentation registered in DWI space, to develop DORIS. Another limitation is the input channel selection that was only based on the Dice score. As we showed in the results, the Dice score did not fully reflect the segmentation quality and a visual human-based segmentation could be worth investigating in the future. However, as mentioned before, this human-based visual inspection, on a large number of subjects and parameters tested during the hyperparameter optimization, would most likely be infeasible in practice.

### 4.4. Impact on tractography

We showed that tractograms based on the DORIS segmentation had longer streamlines than the FSL-Fast Tractoflow equivalent. This difference can be explained by the larger WM volume predicted by DORIS and fewer broken streamlines caused by errors in the tissue classes. Indeed, due to partial volume between the CSF and the WM classified as GM by FSL-Fast, PFT tracking can terminate streamlines in this partial volume, even if this ending point is anatomically incorrect. This advantage of less broken fiber generated by DORIS is also highlighted by the percentage of streamlines in the bundle. This percentage is always higher using DORIS than the FSL-Fast Tractoflow equivalence.

DORIS also seems to be robust to aging white matter hyperintensities, as shown in the corpus callosum example of the ADNI subject. Indeed, the ADNI subject clearly shows a hole in the whole CC based on FSL-Fast. However, using DORIS, the whole CC is not cut and is biased by aging lesions. This result is possible due to the large age range of the training set of DORIS.

### 4.5. Future study

Preliminary results of DORIS-based segmentation used in conjunction with tractography highlight the potential of a native diffusion space segmentation algorithm embedded in the tractography process. More analyses will be made in the near future to quantify the impact of DORIS on connectomics and tractometry.

Another improvement that is possible is to explore different models beyond the denseUnet. Indeed, in this study, we showed that the denseUnet model performs well to do the 10-class segmentation task, which can then improve the bundle reconstruction from tractography using the segmentation output. However, a follow-up study is needed to find the best model to do this task. Due to the big training set we presented, the smallest model architecture as an Unet (Ronneberger et al., 2015) or a fully convolutional network (Long et al., 2015) could work as well as the denseUnet. This study could consist of doing hyperparameter optimizations for each model and then, a quantitative evaluation specifically on the impact of the segmentation on anatomically-constrained tractography reconstructions.

Finally, a new class could be added to segment white matter lesions from aging subjects or subjects with anomalies. This lesion class could permit to do tractometry (Cousineau et al., 2017) analyses directly under the lesion, around it, and in the normal appearing white matter.

## 5. Conclusion

DORIS is the first algorithm to precisely segment voxels of 10 brain tissue classes purely based on diffusion MRI measures. DORIS can work on both single- and multi-shell diffusion MRI acquisition. This study shows the importance of using a big learning set (750 subjects), with a wide age range (from 22 to 90 years old) and variable image quality, to generalize and not bias the learned model. DORIS is fast, accurate, reproducible, and enhances anatomically-constrained tractography producing longer and less anatomically implausible streamlines.

## Data availability statement

Five datasets have been used in this paper, namely Penthera3T, ADNI, PPMI, HCP, and the UKBiobank. Penthera3T, ADNI, PPMI and HCP are publicly accessible and can be found here: https://zenodo.org/record/2602049#.YymIeNVBzJU, https://adni.loni.usc.edu/data-samples/access-data/, https://www.ppmi-info.org/access-data-specimens/download-data and https://www.humanconnectome.org/study/hcp-young-adult/document/1200-subjects-data-release. As for UKBiobank, restrictions apply to the dataset as it cannot be readily available because it is only released by contract. Requests to access the datasets should be directed to https://www.ukbiobank.ac.uk/enable-your-research/apply-for-access.

## Ethics statement

Ethical review and approval was not required for the study on human participants in accordance with the local legislation and institutional requirements. Written informed consent from the patients/participants or patients/participants' legal guardian/next of kin was not required to participate in this study in accordance with the national legislation and the institutional requirements.

## Author contributions

GT and MDe contributed to conception, design of the study, and wrote the first draft of the manuscript. GT and MDu processed the database. ME contributed to the statistical analysis. MZ, SD-G, and CZ contributed to developing part of the code used in the manuscript. P-MJ, MDe, and ME revised the first versions of the manuscript. All authors contributed to manuscript revision, read, and approved the submitted version.

## Funding

This study has been supported by the French government, through the 3IA Côte d'Azur Investments in the Future project managed by the National Research Agency (ANR) with the reference number ANR-19-P3IA-0002. This study has received funding from the European Research Council (ERC) under the European Union's Horizon 2020 research and innovation program (ERC Advanced Grant Agreement No 694665: CoBCoM – Computational Brain Connectivity Mapping).

## Conflict of interest

Authors GT, MDu, CZ, P-MJ, and MDe are employed by the company Imeka Solutions Inc. The remaining authors declare that the research was conducted in the absence of any commercial or financial relationships that could be construed as a potential conflict of interest.

## Publisher's note

All claims expressed in this article are solely those of the authors and do not necessarily represent those of their affiliated organizations, or those of the publisher, the editors and the reviewers. Any product that may be evaluated in this article, or claim that may be made by its manufacturer, is not guaranteed or endorsed by the publisher.

## References

[B1] Avants B. B. Tustison N. Song G. (2009). Advanced normalization tools (ants). Insight J. 2, 1–35. 10.54294/uvnhin

[B2] Aydogan D. B. Shi Y. (2020). Parallel transport tractography. IEEE Trans. Med. Imaging 40, 635–647. 10.1109/TMI.2020.3034038 33104507 PMC7931442

[B3] Battistella G. Najdenovska E. Maeder P. Ghazaleh N. Daducci A. Thiran J.-P.. (2017). Robust thalamic nuclei segmentation method based on local diffusion magnetic resonance properties. Brain Struct. Function 222, 2203–2216. 10.1007/s00429-016-1336-4 27888345 PMC5504280

[B4] Bells S. Cercignani M. Deoni S. Assaf Y. Pasternak O. Evans C.. (2011). “Tractometry-comprehensive multi-modal quantitative assessment of white matter along specific tracts,” in Proceedings of ISMRM, Vol. 678. Montréal, QC, 1.

[B5] Casey B. Cannonier T. Conley M. I. Cohen A. O. Barch D. M. Heitzeg M. M.. (2018). The adolescent brain cognitive development (abcd) study: imaging acquisition across 21 sites. Dev. Cogn. Neurosci. 32, 43–54. 10.1016/j.dcn.2018.03.001 29567376 PMC5999559

[B6] Chamberland M. Raven E. P. Genc S. Duffy K. Descoteaux M. Parker G. D.. (2019). Dimensionality reduction of diffusion mri measures for improved tractometry of the human brain. Neuroimage 200, 89–100. 10.1016/j.neuroimage.2019.06.020 31228638 PMC6711466

[B7] Chamberland M. Whittingstall K. Fortin D. Mathieu D. Descoteaux M. (2014). Real-time multi-peak tractography for instantaneous connectivity display. Front. Neuroinform. 8, 59. 10.3389/fninf.2014.00059 24910610 PMC4038925

[B8] Chen D. Q. Dell'cqua F. Rokem A. Garyfallidis E. Hayes D. J. Zhong J.. (2019). Diffusion weighted image co-registration: investigation of best practices. BioRxiv 864108. 10.1101/864108

[B9] Cheng H. Newman S. Afzali M. Fadnavis S. S. Garyfallidis E. (2020). Segmentation of the brain using direction-averaged signal of dwi images. Magn. Reson. Imaging 69, 1–7. 10.1016/j.mri.2020.02.010 32088291

[B10] Ciritsis A. Boss A. Rossi C. (2018). Automated pixel-wise brain tissue segmentation of diffusion-weighted images via machine learning. NMR Biomed. 31, e3931. 10.1002/nbm.3931 29697165

[B11] Côté M.-A. Girard G. Boré A. Garyfallidis E. Houde J.-C. Descoteaux M. (2013). Tractometer: towards validation of tractography pipelines. Med. Image Anal. 17, 844–857. 10.1016/j.media.2013.03.009 23706753

[B12] Cousineau M. Jodoin P.-M. Garyfallidis E. Côté M.-A. Morency F. C. Rozanski V.. (2017). A test-retest study on parkinson's ppmi dataset yields statistically significant white matter fascicles. Neuroimage Clin. 16, 222–233. 10.1016/j.nicl.2017.07.020 28794981 PMC5547250

[B13] Descoteaux M. (2008). High angular resolution diffusion MRI: from local estimation to segmentation and tractography (Ph.D. thesis). Université Nice Sophia Antipolis.

[B14] Dhollander T. Connelly A. (2016). “A novel iterative approach to reap the benefits of multi-tissue csd from just single-shell (+ b= 0) diffusion mri data,” in Proceedings of ISMRM, Vol. 24. Singapore, 3010.

[B15] Di Tommaso P. Chatzou M. Floden E. W. Barja P. P. Palumbo E. Notredame C. (2017). Nextflow enables reproducible computational workflows. Nat. Biotechnol. 35, 316–319. 10.1038/nbt.3820 28398311

[B16] Dice L. R. (1945). Measures of the amount of ecologic association between species. Ecology 26, 297–302. 10.2307/1932409

[B17] Dumont M. Roy M. Jodoin P.-M. Morency F. C. Houde J.-C. Xie Z.. (2019). Free water in white matter differentiates mci and ad from control subjects. Front. Aging Neurosci. 11, 270. 10.3389/fnagi.2019.00270 31632265 PMC6783505

[B18] Farquharson S. Tournier J.-D. Calamante F. Fabinyi G. Schneider-Kolsky M. Jackson G. D.. (2013). White matter fiber tractography: why we need to move beyond dti. J. Neurosurg. 118, 1367–1377. 10.3171/2013.2.JNS121294 23540269

[B19] Fischl B. (2012). Freesurfer. Neuroimage 62, 774–781. 10.1016/j.neuroimage.2012.01.021 22248573 PMC3685476

[B20] Garyfallidis E. Ocegueda O. Wassermann D. Descoteaux M. (2015). Robust and efficient linear registration of white-matter fascicles in the space of streamlines. Neuroimage 117, 124–140. 10.1016/j.neuroimage.2015.05.016 25987367

[B21] Garza-Villarreal E. A. Alcala-Lozano R. Fernandez-Lozano S. Morelos-Santana E. Dávalos A. Villica na V.. (2021). Clinical and functional connectivity outcomes of 5-hz repetitive transcranial magnetic stimulation as an add-on treatment in cocaine use disorder: A double-blind randomized controlled trial. Biol. Psychiatry 6, 745–757. 10.1016/j.bpsc.2021.01.003 33508499

[B22] Girard G. Whittingstall K. Deriche R. Descoteaux M. (2014). Towards quantitative connectivity analysis: reducing tractography biases. Neuroimage 98, 266–278. 10.1016/j.neuroimage.2014.04.074 24816531

[B23] Glasser M. F. Sotiropoulos S. N. Wilson J. A. Coalson T. S. Fischl B. Andersson J. L.. (2013). The minimal preprocessing pipelines for the human connectome project. Neuroimage 80, 105–124. 10.1016/j.neuroimage.2013.04.127 23668970 PMC3720813

[B24] Groen W. B. Buitelaar J. K. Van Der Gaag R. J. Zwiers M. P. (2011). Pervasive microstructural abnormalities in autism: a dti study. J. Psychiatry Neurosci. 36, 32. 10.1503/jpn.090100 20964953 PMC3004973

[B25] Henschel L. Conjeti S. Estrada S. Diers K. Fischl B. Reuter M. (2020). Fastsurfer-a fast and accurate deep learning based neuroimaging pipeline. Neuroimage 219, 117012. 10.1016/j.neuroimage.2020.117012 32526386 PMC7898243

[B26] Jensen J. H. Helpern J. A. (2010). Mri quantification of non-gaussian water diffusion by kurtosis analysis. NMR Biomed. 23, 698–710. 10.1002/nbm.1518 20632416 PMC2997680

[B27] Jeurissen B. Descoteaux M. Mori S. Leemans A. (2019). Diffusion mri fiber tractography of the brain. NMR Biomed. 32, e3785. 10.1002/nbm.3785 28945294

[B28] Jeurissen B. Szczepankiewicz F. (2021). Multi-tissue spherical deconvolution of tensor-valued diffusion mri. Neuroimage 245, 118717. 10.1016/j.neuroimage.2021.118717 34775006

[B29] Jeurissen B. Tournier J.-D. Dhollander T. Connelly A. Sijbers J. (2014). Multi-tissue constrained spherical deconvolution for improved analysis of multi-shell diffusion mri data. Neuroimage 103, 411–426. 10.1016/j.neuroimage.2014.07.061 25109526

[B30] Kaku A. Hegde C. V. Huang J. Chung S. Wang X. Young M.. (2019). Darts: Denseunet-based automatic rapid tool for brain segmentation. arXiv preprint arXiv:1911.05567. 10.48550/arXiv.1911.05567

[B31] Karan P. Reymbaut A. Gilbert G. Descoteaux M. (2021). Enabling constrained spherical deconvolution and diffusional variance decomposition with tensor-valued diffusion mri. bioRxiv. 10.1101/2021.04.07.438845 35569180

[B32] Koo B.-B. Hua N. Choi C.-H. Ronen I. Lee J.-M. Kim D.-S. (2009). A framework to analyze partial volume effect on gray matter mean diffusivity measurements. Neuroimage 44, 136–144. 10.1016/j.neuroimage.2008.07.064 18775785

[B33] Kreher B. Schneider J. Mader I. Martin E. Hennig J. Il'Yasov K. (2005). Multitensor approach for analysis and tracking of complex fiber configurations. Magn. Reson. Med. 54, 1216–1225. 10.1002/mrm.20670 16200554

[B34] Kurtzer G. M. Sochat V. Bauer M. W. (2017). Singularity: Scientific containers for mobility of compute. PLoS ONE 12, e0177459. 10.1371/journal.pone.0177459 28494014 PMC5426675

[B35] Li H. Liu T. Young G. Guo L. Wong S. T. (2006). “Brain tissue segmentation based on dwi/dti data,” in 3rd IEEE International Symposium on Biomedical Imaging: Nano to Macro, 2006 (Arlington, VA: IEEE), 57–60.

[B36] Little G. Beaulieu C. (2021). Automated cerebral cortex segmentation based solely on diffusion tensor imaging for investigating cortical anisotropy. Neuroimage 237, 118105. 10.1016/j.neuroimage.2021.118105 33933593

[B37] Liu T. Li H. Wong K. Tarokh A. Guo L. Wong S. T. (2007). Brain tissue segmentation based on dti data. Neuroimage 38, 114–123. 10.1016/j.neuroimage.2007.07.002 17804258 PMC2430665

[B38] Long J. Shelhamer E. Darrell T. (2015). “Fully convolutional networks for semantic segmentation,” in Proceedings of the IEEE Conference on Computer Vision and Pattern Recognition (Boston, MA: IEEE), 3431–3440. 10.1109/TPAMI.2016.2572683 27244717

[B39] Maier-Hein K. H. Neher P. F. Houde J.-C. Côté M.-A. Garyfallidis E. Zhong J.. (2017). The challenge of mapping the human connectome based on diffusion tractography. Nat. Commun. 8, 1–13. 10.1038/s41467-017-01285-x 29116093 PMC5677006

[B40] Mandonnet E. Sarubbo S. Petit L. (2018). The nomenclature of human white matter association pathways: proposal for a systematic taxonomic anatomical classification. Front. Neuroanat. 12, 94. 10.3389/fnana.2018.00094 30459566 PMC6232419

[B41] Nie D. Wang L. Adeli E. Lao C. Lin W. Shen D. (2018). 3-d fully convolutional networks for multimodal isointense infant brain image segmentation. IEEE Trans. Cybern. 49, 1123–1136. 10.1109/TCYB.2018.2797905 29994385 PMC6230311

[B42] Paquette M. Gilbert G. Descoteaux M. (2019). Penthera 3T. Zenodo. 10.5281/zenodo.2602049

[B43] Pasternak O. Sochen N. Gur Y. Intrator N. Assaf Y. (2009). Free water elimination and mapping from diffusion mri. Magn. Reson. Med. 62, 717–730. 10.1002/mrm.22055 19623619

[B44] Peled S. Friman O. Jolesz F. Westin C.-F. (2006). Geometrically constrained two-tensor model for crossing tracts in dwi. Magn. Reson. Imaging 24, 1263–1270. 10.1016/j.mri.2006.07.009 17071347 PMC2000805

[B45] Pierpaoli C. Basser P. J. (1996). Toward a quantitative assessment of diffusion anisotropy. Magn. Reson. Med. 36, 893–906. 10.1002/mrm.1910360612 8946355

[B46] Rheault F. (2020). Analyse et reconstruction de faisceaux de la matière blanche (Ph.D. thesis). Université de Sherbrooke.

[B47] Ronneberger O. Fischer P. Brox T. (2015). “U-net: convolutional networks for biomedical image segmentation,” in International Conference on Medical Image Computing and Computer-Assisted Intervention (Springer), 234–241.

[B48] Saygin Z. M. Osher D. E. Augustinack J. Fischl B. Gabrieli J. D. (2011). Connectivity-based segmentation of human amygdala nuclei using probabilistic tractography. Neuroimage 56, 1353–1361. 10.1016/j.neuroimage.2011.03.006 21396459 PMC3102511

[B49] Smith R. E. Tournier J.-D. Calamante F. Connelly A. (2012). Anatomically-constrained tractography: improved diffusion mri streamlines tractography through effective use of anatomical information. Neuroimage 62, 1924–1938. 10.1016/j.neuroimage.2012.06.005 22705374

[B50] Steven A. J. Zhuo J. Melhem E. R. (2014). Diffusion kurtosis imaging: an emerging technique for evaluating the microstructural environment of the brain. Am. J. Roentgenol. 202, W26–W33. 10.2214/AJR.13.11365 24370162

[B51] St-Onge E. Al-Sharif N. Girard G. Theaud G. Descoteaux M. (2021). Cortical surfaces integration with tractography for structural connectivity analysis. Brain Connect. 11, 505–517. 10.1089/brain.2020.0930 34018835

[B52] St-Onge E. Daducci A. Girard G. Descoteaux M. (2018). Surface-enhanced tractography (set). Neuroimage 169, 524–539. 10.1016/j.neuroimage.2017.12.036 29258891

[B53] Theaud G. Fortin D. Morency F. Descoteaux M. (2019). “Brain tumors: a challenge for tracking algorithms,” in 27th Scientific Meeting of the International Society for Magnetic Resonance in Medicine, Montréal, QC.

[B54] Theaud G. Houde J.-C. Boré A. Rheault F. Morency F. Descoteaux M. (2020). Tractoflow: a robust, efficient and reproducible diffusion mri pipeline leveraging nextflow &singularity. Neuroimage 218, 116889. 10.1016/j.neuroimage.2020.116889 32447016

[B55] Tong Q. He H. Gong T. Li C. Liang P. Qian T.. (2020). Multicenter dataset of multi-shell diffusion mri in healthy traveling adults with identical settings. Scientific Data 7, 1–7. 10.1038/s41597-020-0493-8 32461581 PMC7253426

[B56] Tournier J.-D. Calamante F. Connelly A. (2007). Robust determination of the fibre orientation distribution in diffusion mri: non-negativity constrained super-resolved spherical deconvolution. Neuroimage 35, 1459–1472. 10.1016/j.neuroimage.2007.02.016 17379540

[B57] Tournier J.-D. Smith R. Raffelt D. Tabbara R. Dhollander T. Pietsch M.. (2019). Mrtrix3: a fast, flexible and open software framework for medical image processing and visualisation. Neuroimage 202, 116137. 10.1016/j.neuroimage.2019.116137 31473352

[B58] Vanderweyen D. C. Theaud G. Sidhu J. Rheault F. Sarubbo S. Descoteaux M.. (2020). The role of diffusion tractography in refining glial tumor resection. Brain Struct. Function 225, 1413–1436. 10.1007/s00429-020-02056-z 32180019

[B59] Visser E. Keuken M. C. Douaud G. Gaura V. Bachoud-Levi A.-C. Remy P.. (2016). Automatic segmentation of the striatum and globus pallidus using mist: Multimodal image segmentation tool. Neuroimage 125, 479–497. 10.1016/j.neuroimage.2015.10.013 26477650 PMC4692519

[B60] Wang J. Cheng H. Newman S. D. (2020). Sparse representation of dwi images for fully automated brain tissue segmentation. J. Neurosci. Methods 343, 108828. 10.1016/j.jneumeth.2020.108828 32603811

[B61] Wong K. C. Moradi M. Tang H. Syeda-Mahmood T. (2018). “3D segmentation with exponential logarithmic loss for highly unbalanced object sizes,” in International Conference on Medical Image Computing and Computer-Assisted Intervention (Granada: Springer), 612–619.

[B62] Yap P.-T. Zhang Y. Shen D. (2015). “Brain tissue segmentation based on diffusion mri using l 0 sparse-group representation classification,” in International Conference on Medical Image Computing and Computer-Assisted Intervention (Munich: Springer), 132–139. PMC6054460 30035276

[B63] Ye C. Bogovic J. A. Bazin P.-L. Prince J. L. Ying S. H. (2012). “Fully automatic segmentation of the dentate nucleus using diffusion weighted images,” in 2012 9th IEEE International Symposium on Biomedical Imaging (ISBI) (Barcelona: IEEE), 1128–1131.

[B64] Zhang F. Breger A. Cho K. I. K. Ning L. Westin C.-F. O'Donnell L. J.. (2021). Deep learning based segmentation of brain tissue from diffusion mri. Neuroimage 233, 117934. 10.1016/j.neuroimage.2021.117934 33737246 PMC8139182

[B65] Zhang H. Schneider T. Wheeler-Kingshott C. A. Alexander D. C. (2012). Noddi: practical in vivo neurite orientation dispersion and density imaging of the human brain. Neuroimage 61, 1000–1016. 10.1016/j.neuroimage.2012.03.072 22484410

[B66] Zhang W. Li R. Deng H. Wang L. Lin W. Ji S.. (2015). Deep convolutional neural networks for multi-modality isointense infant brain image segmentation. Neuroimage 108, 214–224. 10.1016/j.neuroimage.2014.12.061 25562829 PMC4323729

[B67] Zhang Y. Brady M. Smith S. (2001). Segmentation of brain mr images through a hidden markov random field model and the expectation-maximization algorithm. IEEE Trans. Med. Imaging 20, 45–57. 10.1109/42.906424 11293691

[B68] Zucchelli M. Deslauriers-Gauthier S. Deriche R. (2020). A computational framework for generating rotation invariant features and its application in diffusion mri. Med. Image Anal. 60, 101597. 10.1016/j.media.2019.101597 31810004

