# Supplementary material for: DORIS: A diffusion MRI-based 10 tissue class deep learning segmentation algorithm tailored to improve anatomically-constrained tractography
Source: Front Neuroimaging. 2022 Sep 22;1:917806. doi: 10.3389/fnimg.2022.917806 (PMC10406193; doi:10.3389/fnimg.2022.917806)
Supplement: Supplementary file 1 [file Data_Sheet_1.PDF]

1 SUPPLEMENTARY DATA

Data augmentation ranges for Dense-Unet training

Rotation: [-10, 10]
Shearing: [-0.1, 0.1]
Scaling: [0.9, 1.1]

ANTs registration command for T1 to diffusion space using b0 and FA images.

```
antsRegistration --dimensionality 3 --float 0\
    --output [output,outputWarped.nii.gz,outputInverseWarped.nii.gz]\
    --interpolation Linear --use-histogram-matching 0\
    --winsorize-image-intensities [0.005,0.995]\
    --initial-moving-transform [b0.nii.gz,t1.nii.gz,1]\
    --transform Rigid['0.2']\
    --metric MI[b0.nii.gz,t1.nii.gz,1,32,Regular,0.25]\
    --convergence [500x250x125x50,1e-6,10] --shrink-factors 8x4x2x1\
    --smoothing-sigmas 3x2x1x0\
    --transform Affine['0.2']\
    --metric MI[$b0,$t1,1,32,Regular,0.25]\
    --convergence [500x250x125x50,1e-6,10] --shrink-factors 8x4x2x1\
    --smoothing-sigmas 3x2x1x0\
    --transform SyN[0.1,3,0]\
    --metric MI[b0.nii.gz,t1.nii.gz,1,32]\
    --metric CC[fa.nii.gz,t1.nii.gz,1,4]\
    --convergence [50x25x10,1e-6,10] --shrink-factors 4x2x1\
    --smoothing-sigmas 3x2x1

antsApplyTransforms -v -d 3 -n NearestNeighbor -i wmparc.nii.gz\
    -r b0.nii.gz -t output1Warp.nii.gz -t output0GenericAffine.mat\
    -o wmparc_registered.nii.gz

antsApplyTransforms -v -d 3 -n NearestNeighbor -i aparc+aseg.nii.gz\
    -r b0.nii.gz -t output1Warp.nii.gz -t output0GenericAffine.mat\
    -o aparc_aseg_registered.nii.gz
```

This command is based on the one used in TractoFlow.

2 SUPPLEMENTARY TABLES AND FIGURES

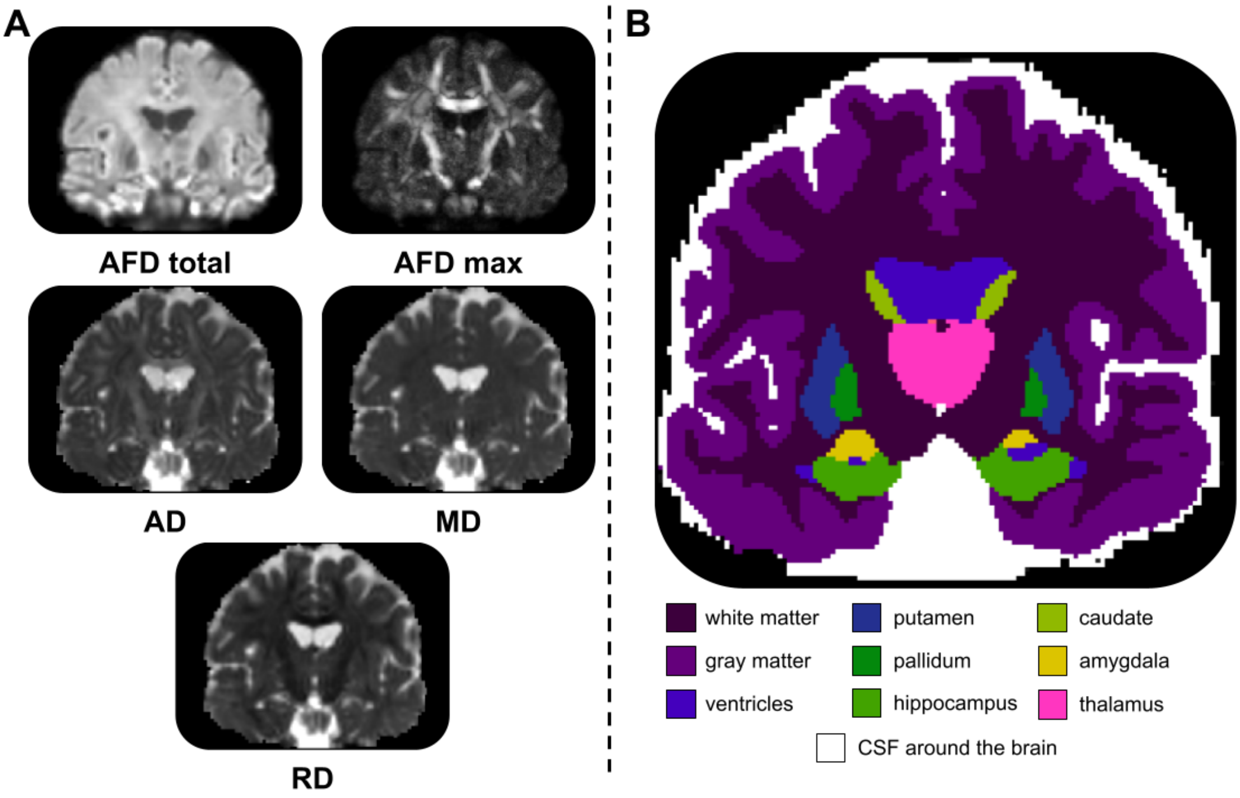

Figure S1. In A, the 5 images used as input channel. In B, the 10 tissue class segmentation.

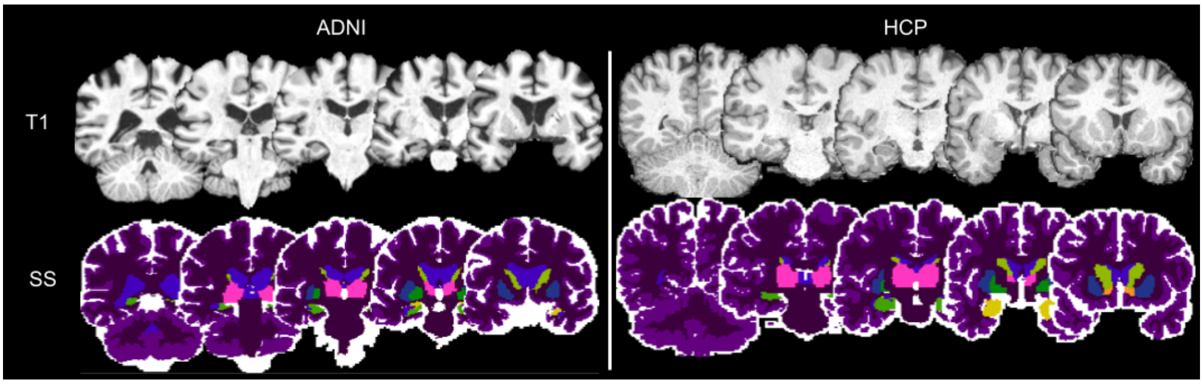

Figure S2. Silver standard image from Freesurfer on ADNI and HCP subjects.

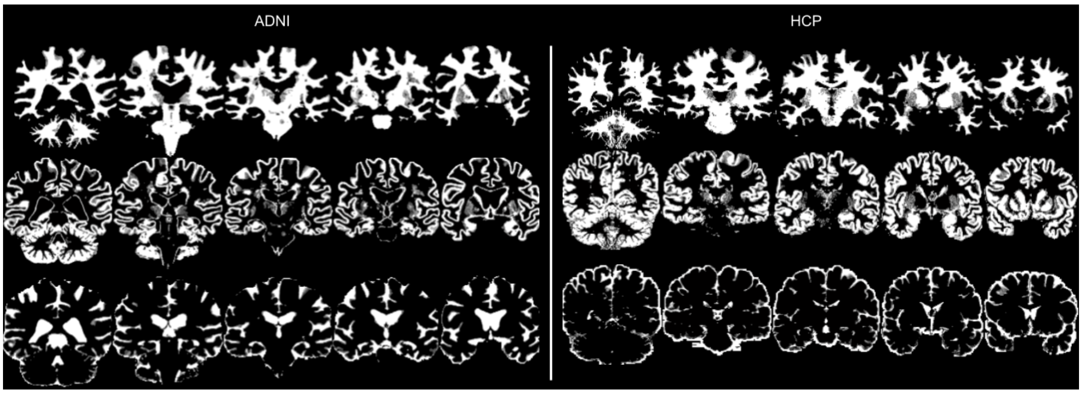

Figure S3. ADNI and HCP subjects segmentation from FSL-Fast

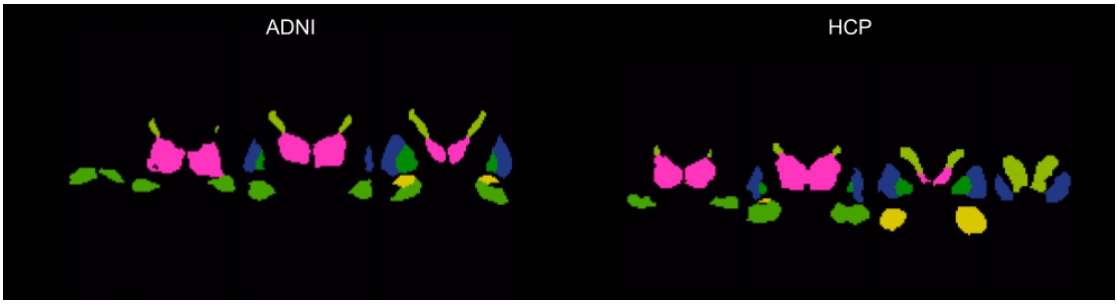

Figure S4. Nuclei mosaic for ADNI and HCP subjects from DORIS.

Table S1. Related t-test for all label volumes between DORIS and the silver standard.

Label	T	p-value
WM	-20.619754316231	1.87002304545186E-31
GM	5.32398808768571	1.16487986447687E-06
Ventricles	-16.3983266370104	1.12776835129094E-25
Putamen	7.24984563457834	4.36694047443652E-10
Pallidum	-17.4565807508637	3.30797195260101E-27
Hyppocampus	-0.819219532230748	0.415441646579405
Caudate	-22.1378407131987	2.48624959519249E-33
Amygdala	-1.4838719980424	0.142331888075401
Thalamus	-6.06896720284198	5.91698289223593E-08

Table S2. Volume (in mm^3) of smallest labels for DORIS and the silver standard

type	label	volume
DORIS	Amygdala	3814 (303)
DORIS	Caudate	7563 (750)
DORIS	Hyppocampus	8432 (578)
DORIS	Pallidum	3100 (559)
DORIS	Putamen	9616 (1137)
DORIS	Thalamus	14137 (1173)
DORIS	Ventricles	22908 (4876)
silver standard	Amygdala	3741 (452)
Silver standard	Caudate	6628 (750)
Silver standard	Hyppocampus	8389 (809)
Silver standard	Pallidum	2322 (358)
Silver standard	Putamen	10040 (1162)
Silver standard	Thalamus	13652 (1068)
Silver standard	Ventricles	20477 (5003)

Table S3. Related t-test for DORIS volumes in test-retest

Label	T	p-value
WM	0.272086349464586	0.790594903814357
GM	-0.606080243692412	0.556761393593017
Ventricles	-0.684811358828159	0.50763586210935
Putamen	0.724749503407634	0.483737254912877
Pallidum	0.885461971013694	0.394851868599488
Hyppocampus	-0.539277357153973	0.600440444292768
Caudate	0.530965542463882	0.605997081871768
Amygdala	-0.346666373623647	0.735382752665228
Thalamus	1.74974259161594	0.107968596843501

Table S4. Related t-test for the silver standard volumes in test-retest

Label	T	p-value
WM	0.0864547032459438	0.932658612248051
GM	-2.05317553446496	0.0646211460904481
Ventricles	-1.07355243429012	0.306009446133064
Putamen	1.03950537071394	0.320879581411232
Pallidum	-1.11117350802823	0.290191085096696
Hyppocampus	-0.175439878929938	0.86392179513451
Caudate	-0.39284293561879	0.701941462917964
Amygdala	-1.6724325678476	0.122609136897853
Thalamus	1.22045802906405	0.24780879896461

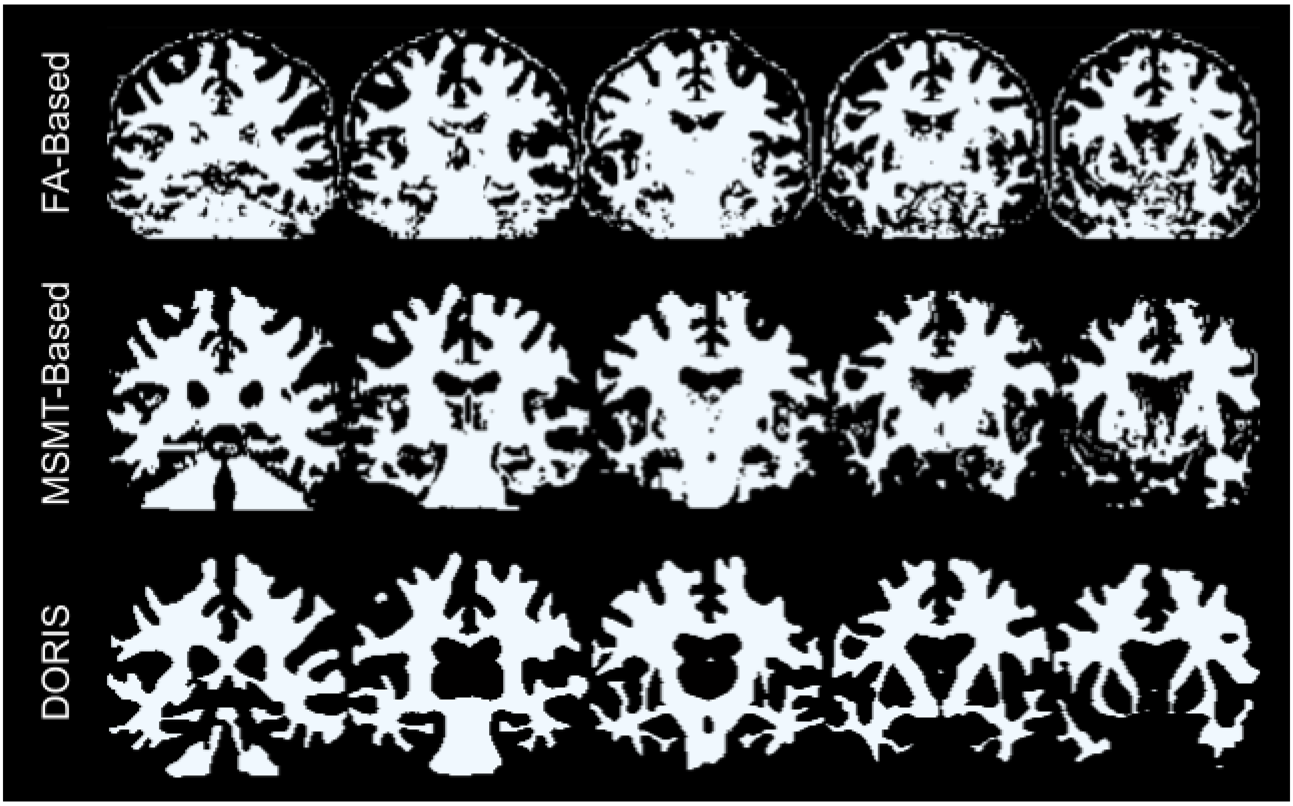

Figure S5. WM mask extracted from FA, MSMT signal fraction and DORIS from a Penthera 3T subject.

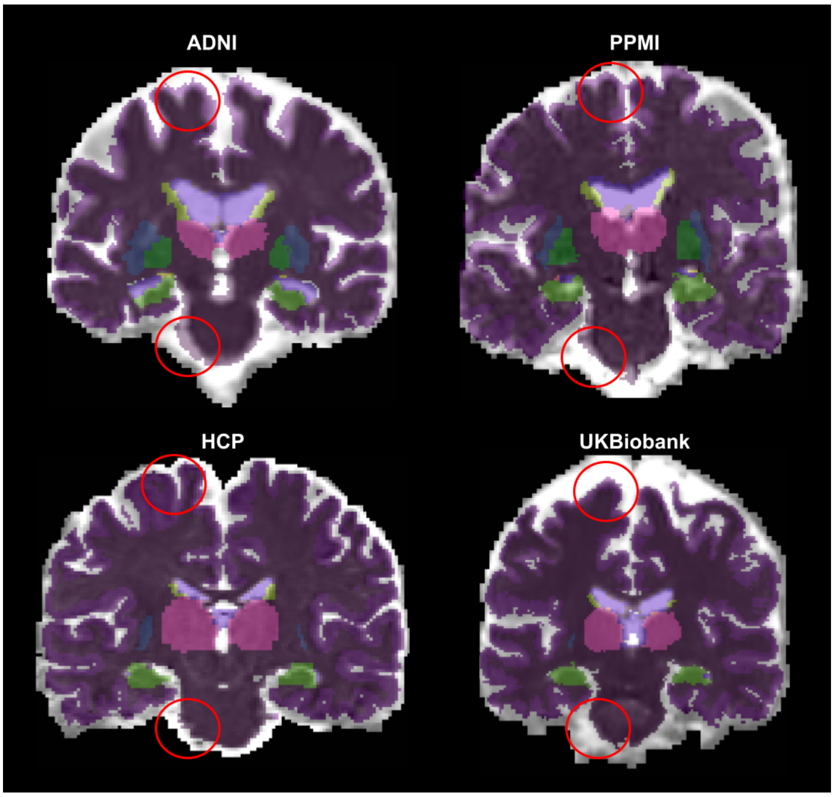

Figure S6. Red circles show segmentation errors in the silver standard that are not present in other subjects. Given a large number of such examples in the training set, one can imagine that the Dense-Unet model will learn to avoid these errors.
